# Identification of Three Campylobacter Lysins and Enhancement of Their Anti-Escherichia coli Efficacy Using Colicin-Based Translocation and Receptor-Binding Domain Fusion

**DOI:** 10.1128/spectrum.04515-22

**Published:** 2023-02-07

**Authors:** Peiqi Liu, Xinying Dong, Xuewei Cao, Qianmei Xie, Xiuqin Huang, Jinfei Jiang, Huilin Dai, Zheng Tang, Yizhen Lin, Saixiang Feng, Kaijian Luo

**Affiliations:** a College of Veterinary Medicine, South China Agricultural University, Guangzhou, China; The Pennsylvania State University

**Keywords:** lysins, antibacterial activity, TRB domains, multidrug resistance, *Campylobacter*, *Escherichia coli*

## Abstract

The emergence of multidrug-resistant Escherichia coli, which poses a major threat to public health, has motivated the development of numerous alternative antimicrobials. Lysins are bacteriophage- and bacterium-derived peptidoglycan hydrolases that represent a new antibiotic treatment targeting bacterial cell walls. However, the bactericidal effect of native lysins on Gram-negative bacteria is restricted by the presence of an outer membrane. Here, we first evaluated the antibacterial activity of three Campylobacter-derived lysins (Clysins) against E. coli. To improve their transmembrane ability and antibacterial activities, six engineered Clysins were constructed by fusing with the translocation and receptor-binding (TRB) domains from two types of colicins (colicin A [TRBA] and colicin K [TRBK]), and their biological activities were determined. Notably, engineered lysin TRBK-Cly02 exhibited the highest bactericidal activity against the E. coli BL21 strain, with a reduction of 6.22 ± 0.34 log units of cells at a concentration of 60.1 μg/mL, and formed an observable inhibition zone even at a dose of 6.01 μg. Moreover, TRBK-Cly02 killed E. coli dose dependently and exhibited the strongest bactericidal activity at pH 6. It also exhibited potential bioactivity against multidrug-resistant E. coli clinical isolates. In summary, this study identified three lysins from Campylobacter strains against E. coli, and the enhancement of their antibacterial activities by TRB domains fusion may allow them to be developed as potential alternatives to antibiotics.

**IMPORTANCE** Three lysins from Campylobacter, namely, Clysins, were investigated, and their antibacterial activities against E. coli were determined for the first time. To overcome the restriction of the outer membrane of Gram-negative bacteria, we combined the TRB domains of colicins with these Clysins. Moreover, we discovered that the Clysins fused with TRB domains from colicin K (TRBK) killed E. coli more effectively, and this provides a new foundation for the development of novel bioengineered lysins by employing TRBK constructs that target outer membrane receptor/transport systems. One of the designed lysins, TRBK-Cly02, exhibited potent bactericidal efficacy against E. coli strains and may be used for control of multidrug-resistant clinical isolates. The results suggest that TRBK-Cly02 can be considered a potential antibacterial agent against pathogenic E. coli.

## INTRODUCTION

The widespread emergence and dissemination of diarrheagenic Escherichia coli strains, which can be found throughout the food chain, pose serious threats to public health, especially due to the increasing multidrug resistance of the bacteria (1–3). In the past decade, E. coli isolates from hospitals and retail food locations in China with multidrug resistance to currently available drugs, including ampicillin, cefotaxime, ciprofloxacin, tetracycline, and florfenicol, have been reported (4–6). Antimicrobial resistance (AMR) is an inevitable evolutionary outcome since bacteria develop genetic mutations to naturally prevent lethal selection pressure. However, the probability of AMR occurring is increasing for various reasons, including abuse of antibiotics, widespread agricultural usage of antibiotics, and improper prescribing of antibiotics (7–9), and these situations with potentially serious consequences pervade our daily lives. In a 2019 comprehensive study of global antimicrobial-resistant bacterial issues, E. coli was described as a high-priority pathogen accounting for 929,000 deaths due to AMR (10). Moreover, in addition to contributing to the growing phenomenon of bacterial resistance, the challenge of antibiotic residues in food and environments may pose high health risks to humans and animals. Antibiotic residues may change the microbial composition in humans and animals and promote the emergence of antimicrobial-resistant bacteria, which harbor antimicrobial resistance genes that will spread between the same or different pathogenic bacteria (11–13). Furthermore, antibiotic residues in food can have counterproductive effects on humans by detrimentally disrupting the gut microbiome and affecting organ systems, leading to disease and even mortality (14, 15). Thus, there is an urgent need for novel therapeutics to combat high-threat pathogens.

Recently, numerous new antibacterial strategies, such as silver nanoparticles, bacteriophages and phage-encoded enzymes, have been introduced in the postantibiotic era as developing new antibiotics is difficult and studying the efficacy and safety of antibiotic agents is time and effort intensive (16–18). Lysins, a class of peptidoglycan-hydrolyzing enzymes, have several advantages over antibiotics, including the ability to rapidly lyse bacteria on contact with the bacterial cell wall. Numerous lysins exhibit more impressive potencies than antibiotics due to their antibiofilm functions *in vitro* (19–21). Endolysins, derived from phages, are involved in the lytic period of phages and can create holes in the internal cell wall of bacteria by digesting peptidoglycan (PG), thereby thoroughly lysing the bacteria (22). Autolysins, derived from bacteria, perform indispensable roles in cell wall metabolism, including the remodeling of PG during the bacterial growth cycle to enable the cellular structure to remain intact and maintain normal microbial function (23, 24). For instance, the major autolysin of Staphylococcus aureus, called Atl, functions during cell expansion and division (25), thereby minimizing the possibility of bacterial resistance to it. Additionally, lysins have not been reported to potentially impact bacterial evolution because of highly conserved PG structures. Recently, various studies focused on overcoming the shortcomings of antibiotics have explored the use of lysins. So far, numerous studies have been conducted regarding the exogenous expression and bactericidal properties of lysins, demonstrating good lytic activity of lysins against Staphylococcus spp. and Acinetobacter baumannii (26–28). Some lysins exhibit broad-spectrum antibacterial activity. For example, LysAm24, LysECD7, and LysSi3, derived from *Myoviridae* bacteriophages, exhibit antibacterial activity against clinical isolates of Pseudomonas aeruginosa, A. baumannii, Klebsiella pneumoniae, E. coli, and Salmonella (29). Ramesh et al. demonstrated that Gp105, a putative endolysin from Enterobacter phage myPSH1140, could effectively kill numerous clinical isolates, such as E. coli, Klebsiella pneumoniae, Enterobacter cloacae, and A. baumannii strains (30). Additionally, some clinical trials have used lysins to treat staphylococcal infection in animal infection models (31, 32). In a previous study, exebacase (also named CF-301 and which is an antistaphylococcal lysin encoded within a prophage of the Streptococcus suis genome [33, 34]) was combined with antibiotics to treat Staphylococcus aureus endocarditis, and no ensuing hypersensitivity reactions were observed (35). Wire et al. demonstrated that LSVT-1701 (SAL200), a recombinantly produced and S. aureus bacteriophage-encoded lysin (36), is harmless and helpful in treating patients with Staphylococcus aureus bacteremia and infective endocarditis (37).

Lysins are widely recognized as safe and highly effective antimicrobial agents; however, their killing effect on Gram-negative bacteria is less notable than that on Gram-positive bacteria. This is because the outer membrane (OM) of Gram-negative bacteria acts as a barrier preventing lysins from approaching the PG, thereby protecting the bacteria from the killing effect (38, 39). Numerous previous studies were initiated from the perspective of genetic engineering, aiming to exert the strongest lytic activity against Gram-negative bacteria by engineering lysins with OM-destabilizing peptides, fusing different lytic enzymes together, or fusing lysins with protein domains that target OM receptor/transport systems (40–43). Colicins belong to the last category. Colicins are bacteriocins that confer a competitive advantage to bacteria in survival. Colicins generally comprise three structural domains, namely, an N-terminal translocation (T) domain, a central receptor-binding (RB) domain targeting cell surfaces, and a C-terminal cytotoxic (C) domain (44). Both colicin A and colicin K are categorized as A-group colicins, and they kill other bacteria in the environment by crossing the OM through the Tol system (45). When the RB domain interacts with the BtuB transporter (or the Tsx receptor for colicin K), the T domain becomes exposed to the OmpF porin. When colicins enter the periplasmic space, the Tol system is triggered and facilitates the entry of colicins into the bacterial cytoplasm. Subsequently, the C region exerts enzymatic activity interfering with the normal activities of bacteria (46–48). According to reports regarding the import mechanism of colicins and antibacterial activity of some bioengineered lysocins (bioengineered lysin-bacteriocin fusion molecules) (49–51), the translocation and receptor-binding (TRB) domains of colicins are believed to be the keys that open the gate of the OM, thereby assisting lysins in crossing it and entering the periplasmic space to cleave PG.

In this study, three Campylobacter-derived lysins (Clysins) were selected from the GenBank database and fused with the TRB domains of colicin A (TRBA) or colicin K (TRBK) at the N terminus to verify the bactericidal activity of the lysins and possibly develop a novel potent antibacterial agent against Gram-negative bacteria. The antibacterial efficacies of the Clysins and TRB-Clysins were evaluated *in vitro* to determine if the three lysins possess antibacterial properties and can be transported through the OM using TRB domains evolved by bacteria.

## RESULTS

### Expression of Clysins in a prokaryotic E. coli system.

**FIG 1:**
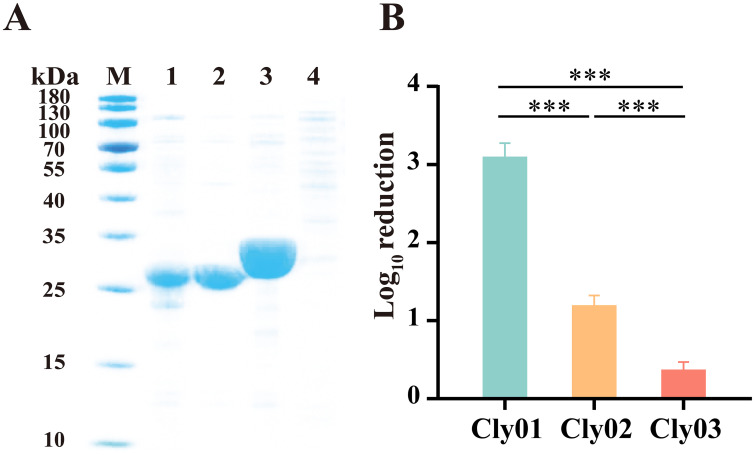
Expression and antibacterial bioactivity of Cly01, Cly02, and Cly03. (A) SDS-PAGE analysis of the purified proteins. Lanes: M, 180-kDa prestained protein marker; 1, Cly01; 2, Cly02; 3, Cly03; 4, Escherichia coli BL21(DE3) competent cells without plasmids. (B) Antibacterial bioactivity of 1 μM lysins against logarithmic-phase E. coli BL21 in 10 mM phosphate-buffered saline (pH 7.4) measured in log_10_ units. The error bars show the standard deviation. Data were replicated for three independent experiments and analyzed using one-way analysis of variance. *, *P* < 0.05; **, *P* < 0.01; ***, *P* < 0.001.

The codon-optimized DNA sequences of three Clysins (Cly01, Cly02, and Cly03) were synthesized and cloned into pET-28α(+) vectors transformed in the E. coli BL21(DE3) strain. Clysin proteins were purified via affinity Ni-nitrilotriacetic acid (NTA) resin. Finally, we visualized three Clysins using sodium dodecyl sulfate-polyacrylamide gel electrophoresis (SDS-PAGE) analysis. The SDS-PAGE showed a single band in three lanes between the 25- and 35-kDa ladders, and the molecular weights of Cly01, Cly02, and Cly03 were similar to their predicted molecular weights (24.8, 25.6, and 32.3 kDa, respectively), thereby confirming that the three Clysins were successfully expressed (Fig. 1A).

### Enhancement of the bactericidal ability of the Clysins when linked to TRBA.

**FIG 2:**
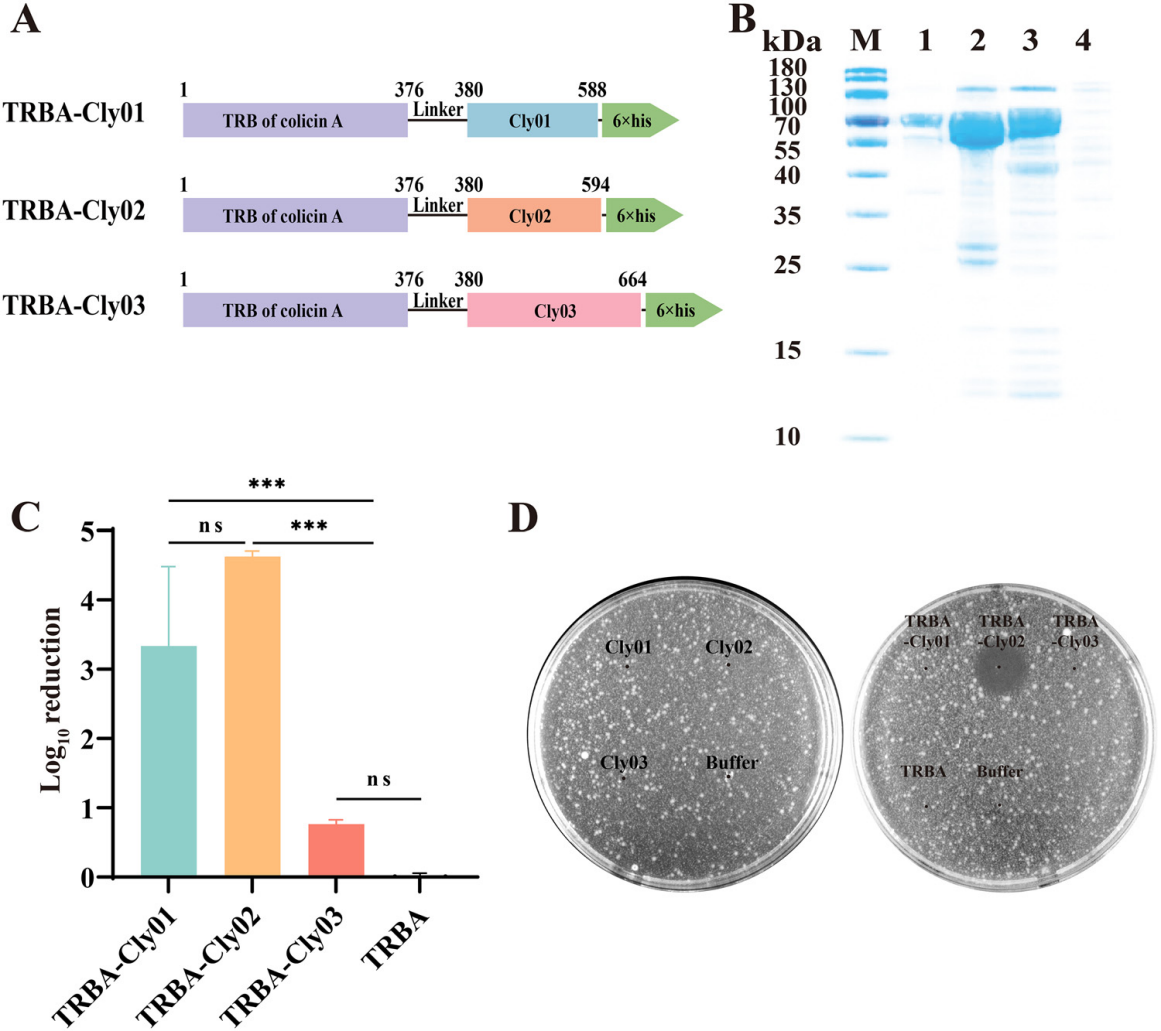
Antibacterial activities of the TRBA-Clysins. (A) Visual representation of the bioengineered TRBA-Clysins. TRBA was fused with the Clysins (linker GGGGS). (B) SDS-PAGE analysis of TRBA-Clysins. Lanes: M, 180-kDa prestained protein marker; 1, TRBA-Cly01; 2, TRBA-Cly02; 3, TRBA-Cly03; 4, Escherichia coli BL21(DE3) competent cells without plasmids. (C) Bacterial bioactivity of 1 μM TRBA-Clysins against logarithmic-phase E. coli BL21 in 10 mM phosphate-buffered saline (pH 7.4) depicted in log_10_ units. The error bars show the standard deviation. Data were replicated for three independent experiments and analyzed using one-way analysis of variance. ns, not significant; *, *P* < 0.05; **, *P* < 0.01; ***, *P* < 0.001. (D) Plate lysis assay. Here, 0.1 nmol each of Clysins and TRBA-Clysins was spotted onto the double-layer plate containing E. coli BL21. The tests were performed in triplicate.

The killing efficiency of 1 μM Clysins (24.8 μg/mL for Cly01, 25.6 μg/mL for Cly02, and 32.2 μg/mL for Cly03) against E. coli BL21 was evaluated using an antibacterial activity test. Cly02 and Cly03 exhibited limited killing efficiency, reducing 1.20 ± 0.12 and 0.37 ± 0.10 log units of E. coli BL21, respectively (Fig. 1B). Surprisingly, Cly01 exhibited the highest antimicrobial activity against E. coli BL21 among the three monomeric Clysins, with a reduction of 3.10 ± 0.17 log units of E. coli BL21. According to previous studies, the OM of Gram-negative bacteria prevents Clysins from entering the periplasmic space, thereby preventing the cell wall from being cleaved to a considerable extent (38, 39). Therefore, TRBA was derived from colicin A (GenBank accession no. WP_008323639.1) by domain analysis and fused with the three Clysins for expression and purification, yielding TRBA-Cly01, TRBA-Cly02, and TRBA-Cly03. Figure 2A depicts the modular structure of the TRBA-Clysins. TRBA-Cly01, TRBA-Cly02, and TRBA-Cly03 have apparent molecular weights of 64.9, 65.8, and 72.6 kDa, respectively, and were visualized using SDS-PAGE analysis (Fig. 2B).

Similarly, we conducted an antibacterial activity test on the TRBA-Clysins at a concentration of 1 μM (64.9 μg/mL for TRBA-Cly01, 65.8 μg/mL for TRBA-Cly02, and 72.6 μg/mL for TRBA-Cly03). Contrary to Cly02, TRBA-Cly02 exhibited a notable bactericidal activity by killing 4.63 ± 0.08 log units of E. coli BL21, which is ~3.86-fold the level killed by Cly02 (Fig. 2C). However, TRBA-Cly01 and TRBA-Cly03 exhibited slightly higher antibacterial activities than their parental lysins, with reductions of 3.34 ± 1.15 and 0.76 ± 0.06 log units of E. coli BL21, respectively. Notably, TRBA-Cly02 exhibited the strongest bactericidal activity against E. coli BL21 in 10 mM phosphate-buffered saline (PBS) among the three TRBA-Clysins, replacing the former position of Cly01 with regard to the parental lysins. As expected, TRBA did not affect the survival of E. coli BL21.

A plate lysis assay was performed to compare the inhibitory bioactivities of these six lysins against E. coli BL21 in a lysogeny broth (LB) medium (Fig. 2D). Notably, similar results were obtained in a spot-on-lawn assay. No clear zone was observed, except in the TRBA-Cly02-spotted area, which formed a lysis zone with a diameter of ~1.47 cm. Hence, the germicidal functions of the Clyins were enhanced when fused with the TRBA. Remarkably, TRBA-Cly02 exhibited antiseptic activity against E. coli BL21 on the LB medium. However, the results of the antibacterial activity test of the TRBA-Clysins against Salmonella demonstrated that TRBA-Clysins exhibited no bactericidal activity against Salmonella spp. (see Fig. S1 in the supplemental material).

### Remarkable lytic activity of TRBK-Cly02.

**FIG 3:**
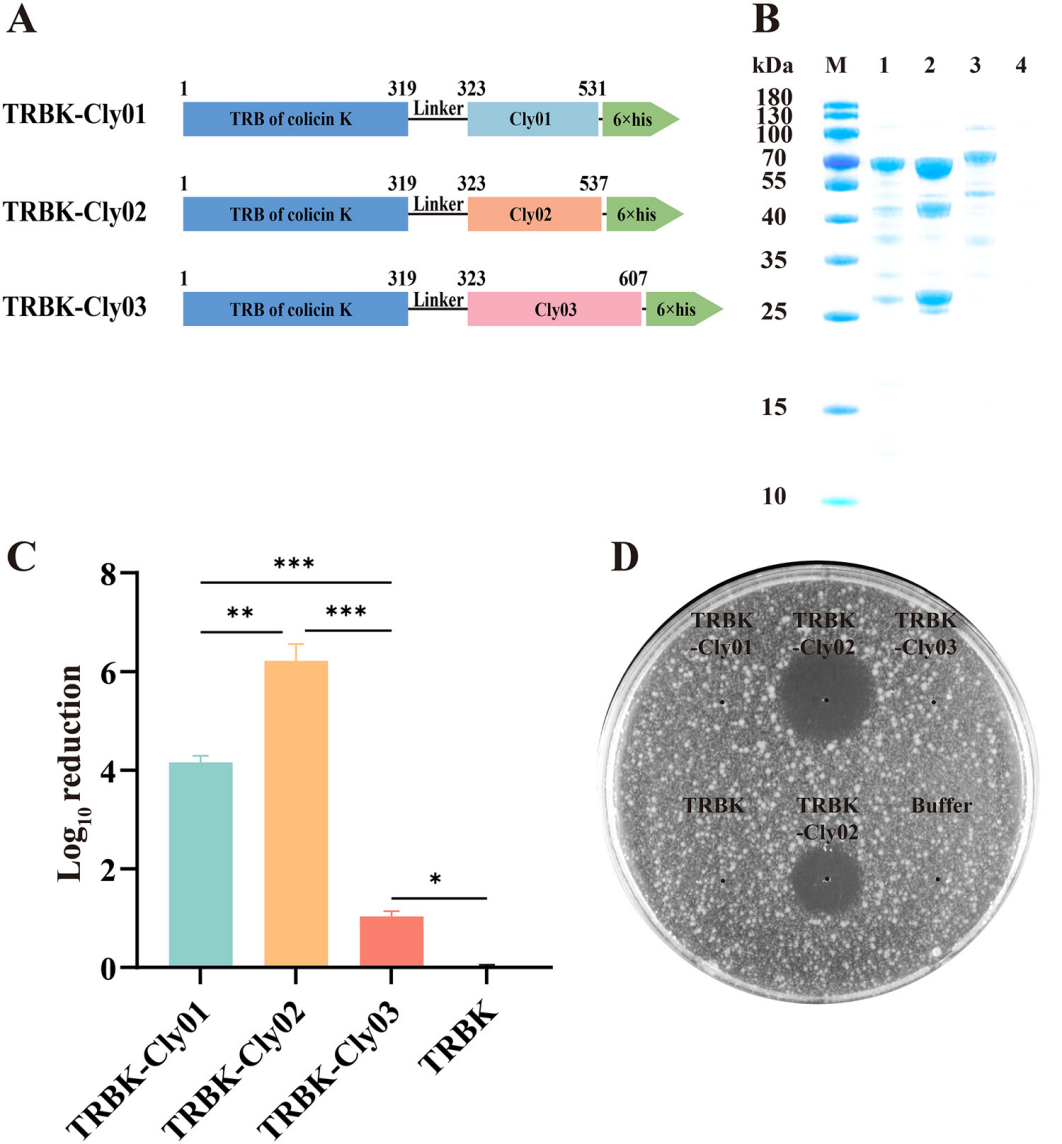
Antibiotic bioactivity of the TRBK-Clysins. (A) Visual representation of the bioengineered TRBK-Clysins. TRBK was fused with the Clysins (linker GGGGS). (B) SDS-PAGE analysis of TRBK-Clysins. Lanes: M, 180-kDa prestained protein marker; 1, TRBK-Cly01; 2, TRBK-Cly02; 3, TRBK-Cly03; 4, Escherichia coli BL21(DE3) competent cells without plasmids. (C) Antibiotic bioactivity of 1 μM TRBK-Clysins against logarithmic-phase E. coli BL21 in 10 mM phosphate-buffered saline (pH 7.4) depicted in log_10_ units. The error bars show the standard deviation. The data were replicated for three independent experiments and analyzed using one-way analysis of variance. *, *P* < 0.05; **, *P* < 0.01; and ***, *P* < 0.001. (D) Plate lysis assay. Here, 0.1 nmol each of TRBA-Cly02, TRBK-Clysins, and TRBK was spotted onto the double-layer plate containing E. coli BL21. The tests were performed in triplicate.

In order to help Clysins exhibit bactericidal activity against Salmonella spp., the bacteriocin colicin K (GenBank accession no. EHY5305768.1), derived from E. coli and which also exists in Salmonella, was found to be similar to colicin A via alignment analysis. We speculated that colicin K may have the ability to enter Salmonella; therefore, we reconstituted bioengineered Clysins using the TRB domain from colicin K (TRBK). TRBK was fused with Cly01, Cly02, and Cly03 using a method similar to that for TRBA (Fig. 3A). Figure 3B presents the resulting three proteins, named TRBK-Cly01, TRBK-Cly02, and TRBK-Cly03, having apparent molecular weights of 59.3, 60.1, and 66.8 kDa, respectively.

First, an antibacterial activity test on the TRBK-Clysins against Salmonella spp. was performed, which demonstrated the futility of TRBK-Clysins against Salmonella spp. (data not shown). Interestingly, we found that TRBK-Clysins have potent bactericidal effects on E. coli BL21, which killed even stronger than TRBA-Clysins. The number of bacteria in all the experimental groups decreased after E. coli BL21 was suspended following incubation with 59.3 μg/mL TRBK-Cly01, 60.1 μg/mL TRBK-Cly02, and 66.8 μg/mL TRBK-Cly03 (1 μM TRBK-Clysins) for 16 h, respectively (Fig. 3C). Particularly, TRBK-Cly02 exhibited the most remarkable bactericidal activity, with a reduction of 6.22 ± 0.34 log units of E. coli BL21, which is ~5.18-fold higher than that of Cly02. However, TRBK-Cly01 and TRBK-Cly03 exhibited somewhat higher bactericidal activities than their parental lysins, reducing 4.16 ± 0.13 and 1.04 ± 0.10 log units of E. coli BL21, respectively. As expected, TRBK did not affect the survival of E. coli BL21 as well.

We speculated if TRBK had the same ability to enhance the lytic activity of the Clysins in the LB medium as TRBA. Consequently, 0.1 nmol each of TRBA-Cly02, TRBK-Clysins, and TRBK was spotted onto a double-layer plate containing E. coli BL21. A buffer without protein was used as a negative control. No bacteria were suppressed in the buffer and TRBK groups (Fig. 3D). As expected, the inhibition region was clearly noticeable under TRBK-Cly02 and TRBA-Cly02 treatment. TRBK-Cly02 exhibited a larger inhibition zone than TRBA-Cly02, with a diameter of ~1.94 cm. Therefore, the TRBK module enhances activity of the Clysins and performed better than the TRBA module, thereby rendering to TRBK-Cly02 remarkable lytic efficacy.

### Efficient killing of E. coli BL21 strains by chimeric TRBK-Cly02 under slightly acidic conditions.

**FIG 4:**
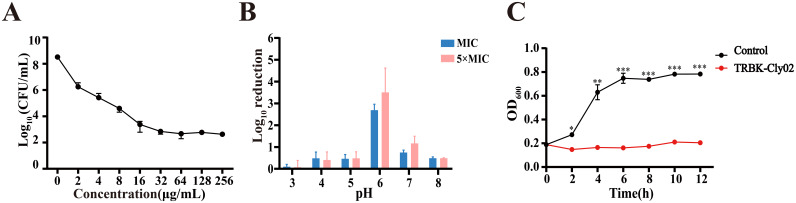
Bactericidal properties of TRBK-Cly02. (A) Dose-dependent bactericidal activity of different concentrations of TRBK-Cly02 in phosphate-buffered saline (PBS) (pH 7.4) against E. coli BL21. (B) Effects of pH on the MIC/5× MIC activity of TRBK-Cly02 against E. coli BL21 in PBS. (C) Time-dependent inhibition curve of 32 μg/mL TRBK-Cly02 against E. coli BL21 in 12 h. The data were analyzed by the paired-sample *t* test. The data are presented as the mean ± standard deviation of results from triplicate independent experiments. *, *P* < 0.05; **, *P* < 0.01; ***, *P* < 0.001.

The above-mentioned results indicate that TRBK-Cly02 exhibits a strong antibacterial potential against E. coli BL21. To further verify the biochemical characteristics of TRBK-Cly02, we explored its bacteriolytic activity using different pH values in 10 mM PBS at a MIC of 0.65 μg/mL and 5× MIC of 3.25 μg/mL. TRBK-Cly02 exhibited high-efficiency antibacterial activity at pH 6, achieving bacterial reductions of 2.69 ± 0.27 and 3.50 ± 1.12 log CFU/mL at values of MIC and 5× MIC, respectively (Fig. 4B). Cly02 exerts its greatest potential to kill E. coli BL21 strains at pH 6, which is the same as TRBK-Cly02 (Fig. S2). Meanwhile, TRBK-Cly02 exhibited limited antimicrobial bioactivity at other pH values, with bacterial reductions of <0.8 and <1.2 log units at the MIC and 5× MIC, respectively. However, the same dose of TRBK-Cly02 barely displayed any bacteriolytic bioactivity at pH 3. Overall, the optimum pH for TRBK-Cly02 was determined to be 6, with activity demonstrated at pH 4 to 8.

### Dose-dependent killing and growth inhibition efficiency of TRBK-Cly02 against E. coli BL21.

E. coli BL21 was incubated with gradient concentrations of TRBK-Cly02 at pH 7.4 for 16 h to determine the activity of TRBK-Cly02. The log CFU reduction is displayed in Fig. 4A. The dose-dependent activity of TRBK-Cly02 became more distinct with the increase in concentration and the killing of E. coli BL21 became more pronounced. Specifically, ~5.92 log units of cells were reduced after treatment with *≥*32 μg/mL TRBK-Cly02 at 37°C for 16 h. Even 2 μg/mL of TRBK-Cly02 destroyed 2.60 ± 0.58 log units of E. coli BL21.

A turbidity assay was performed to evaluate the optical density at 600 nm (OD_600_) of E. coli BL21 treated with TRBK-Cly02 or buffer without proteins to assess the growth inhibition ability of TRBK-Cly02 during the growth of cells. The OD_600_ of E. coli BL21 was 0.2 before the assay. Compared with the negative control, the TRBK-Cly02-treated cells were persistently suppressed within 12 h (Fig. 4C). Throughout this experiment, the OD_600_ of the negative control increased from 0.2 to 0.78, and the bacteria reached the stationary phase at 6 h. However, the OD_600_ of TRBK-Cly02-treated cells fluctuated around 0.2 and decreased slightly following 2 h of incubation. Therefore, TRBK-Cly02 could persistently control E. coli BL21 at a low dosage.

### Visualizing the mechanism of bioengineered TRBK-Cly02 anti-E. coli ability.

**FIG 5:**
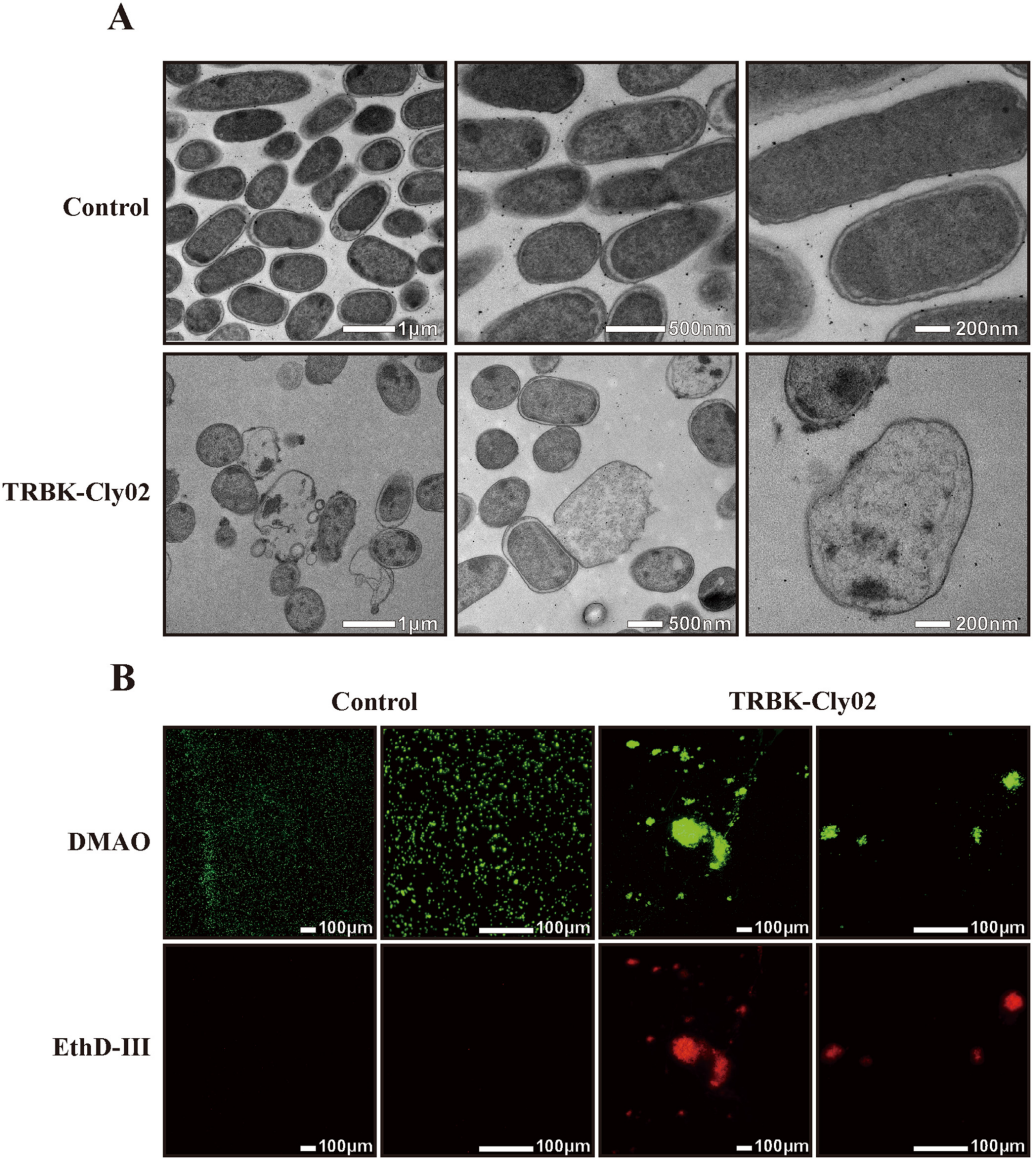
Microscopic observation of the bacteria. (A) Transmission electron microcopy observation of E. coli BL21 cells with and without 32 μg/mL TRBK-Cly02 treatment; (B) fluorescence images of live and dead E. coli BL21 cells stained with DMAO and EthD-III. A size bar is shown in each panel.

To gain an improved understanding of the killing effect of TRBK-Cly02 on E. coli BL21, the cells were incubated with 32 μg/mL of TRBK-Cly02 for 16 h before being prepared for transmission electron microscopy (TEM) analysis. Cells diluted in the buffer were routinely used as a negative control. As shown in Fig. 5A, the TEM morphology of the E. coli cells from the test group showed extreme damage, with cracked and ruptured cell surfaces and microscopic particles or precipitates near the damaged cells. Moreover, chromatin condensations were observed to be wrapped into apoptotic-like bodies. However, the cell surfaces of the control group were smooth with intact integrity, a multilayered cell structure comprising an OM, a PG layer in the periplasmic space, and cytoplasmic membrane.

We examined the E. coli BL21 cells in the test and control groups using a live and dead bacterial staining kit and fluorescence microscopy to demonstrate these outcomes via an independent method (Fig. 5B). The strong red fluorescence signals of the test group were observed in a Cy3 channel, suggesting that the bacterial membrane was damaged by TRBK-Cly02. Conversely, cells in the control group exhibited bright green fluorescence in a fluorescein isothiocyanate (FITC) channel and their red fluorescent signal was very weak to be observed in a Cy3 channel. This suggested that the cells in the control group were viable. The above results confirm that bioengineered TRBK-Cly02 exhibits a significant ability to severely damage E. coli BL21 strains.

### Potential broad-spectrum bioactivity of TRBK-Cly02 against E. coli.

**FIG 6:**
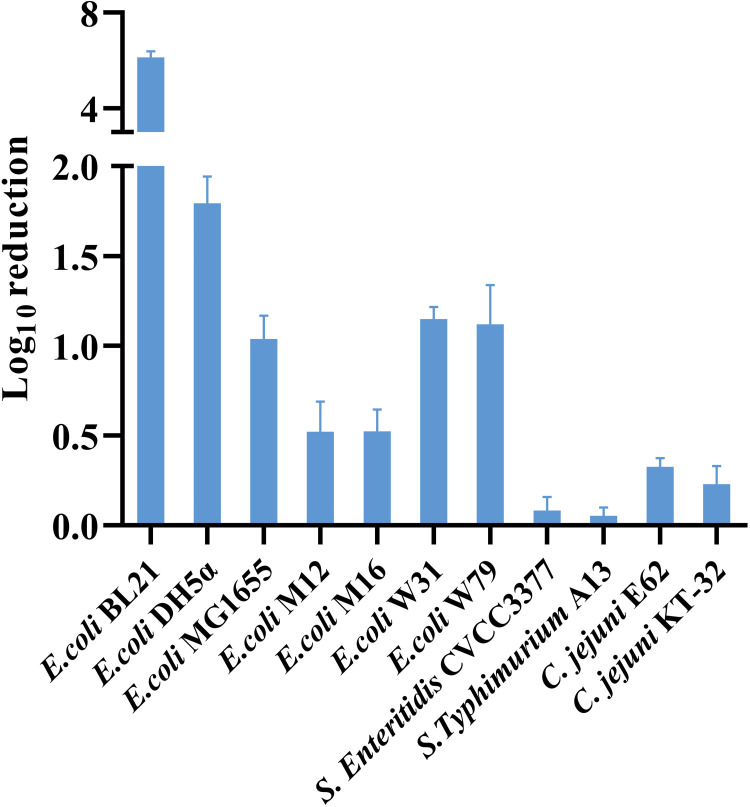
Antibacterial spectrum of TRBK-Cly02 against E. coli, Salmonella, and Campylobacter jejuni strains. The antibacterial activities of TRBK-Cly02 were assessed in PBS (pH 6.0) with 5 mM EDTA and are indicated by log_10_ reduction of cells. Data are presented as the mean ± standard deviation of results from triplicate independent experiments.

The spectrum activity of TRBK-Cly02 under the concentration of 60.1 μg/mL was assessed in PBS (pH 6) with 5 mM EDTA against a group of 11 Gram-negative bacteria, including E. coli, Salmonella spp., and Campylobacter jejuni. TRBK-Cly02 efficiently killed five out of seven E. coli strains (BL21, DH5α, MG1655, W31, and W79), reducing >1 log unit of cells (Fig. 6). However, E. coli MG1655, W31, and W79 are insensitive to Cly02 (Fig. S3). Particularly, TRBK-Cly02 exhibited the strongest killing efficiency against E. coli BL21 and DH5α strains, with reductions of 6.13 ± 0.25 and 1.79 ± 0.15 log units, respectively. The killing bioactivity of TRBK-Cly02 was also observed against two clinical isolates, E. coli W31 and W79, with bacterial reductions of 1.15 ± 0.07 and 1.12 ± 0.22 log units, respectively. TRBK-Cly02 exerted only limited antibacterial activity against C. jejuni E62 and C. jejuni KT-32, with reductions of 0.33 ± 0.05 and 0.23 ± 0.10 log CFU, respectively. However, it exhibited no bactericidal activity against Salmonella. This suggests that TRBK-Cly02 has potential broad-spectrum bioactivity against E. coli.

### Prediction that the mechanism of Clysins cleaves bacterial cell walls.

**FIG 7:**
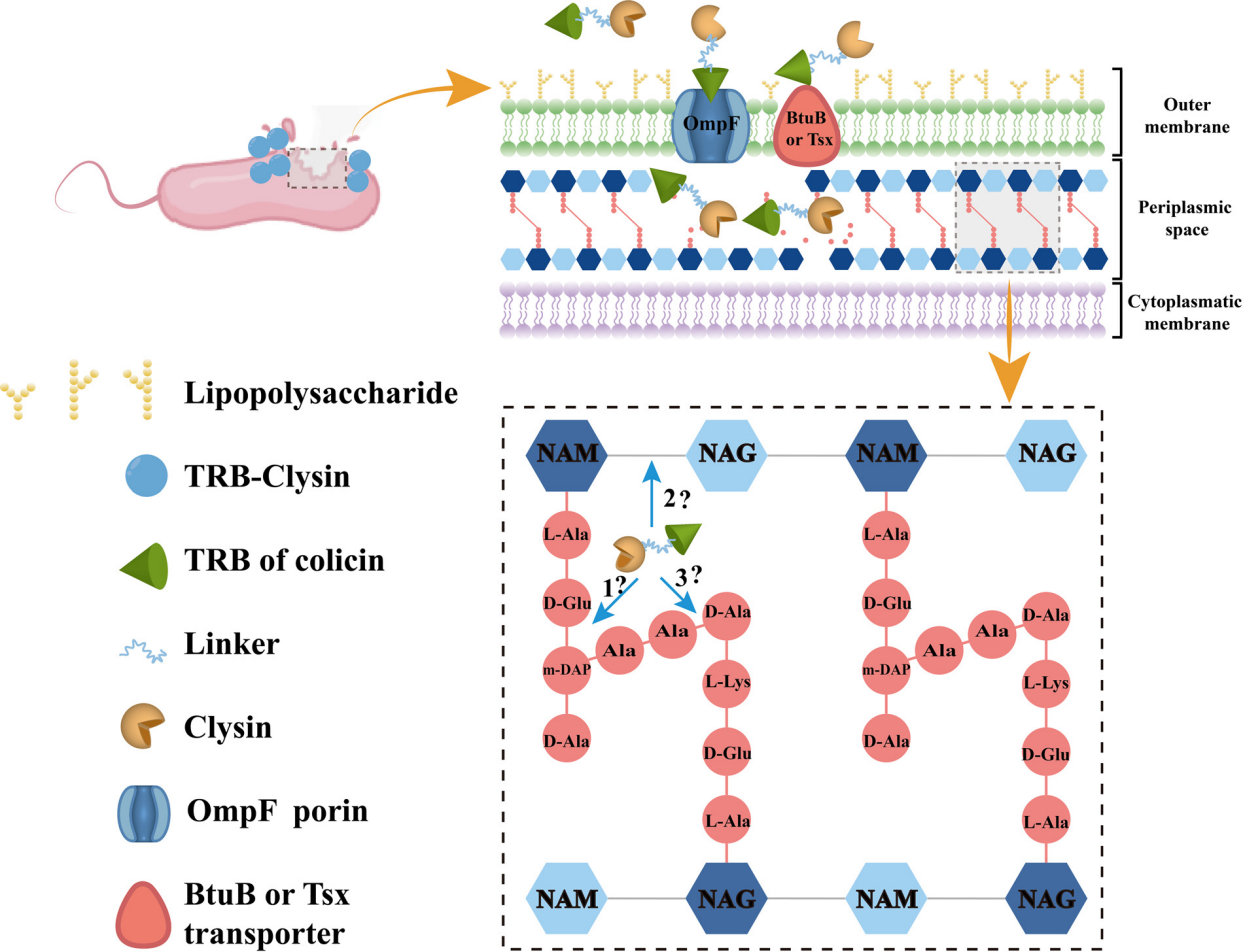
Schematic of the predicted mechanism by which Clysins cleave bacterial cell walls. The parts of the process are shown as follows: (part 1) Cly01, which probably hydrolyzed the peptide bond between d-Glu and mDAP; (part 2) Cly02, which may have cleaved the glycan chains between *N*-acetylmuramic acid (NAM) and *N*-acetylglucosamine (NAG); (part 3) Cly03, which may prefer to hydrolyze the peptide bond between the d-Ala residue and l-Ala in the peptide cross bridge.

To further elucidate the bactericidal action of Clysins on E. coli, we aligned the amino acid sequences of the cell wall lytic enzymes with the reported protein sequences in the Protein Data Bank and UniProt Knowledgebase; the amino acid sequence of Cly02 shares 38.19% identity with a protein component of lambda lysozyme (LaL) (PDB no. 1D9U) from *Lambdavirus*, 36.81% identity with LaL (PDB no. 1AM7), and 23.33% identity with *Escherichiavirus* T4 endolysin (PDB no. 6U0E). Cly03 shares 30.17% identity with a solution structure (PDB no. 2LS0) of zoocin A endopeptidase (data not shown). Cly02 was aligned with a similar cell wall lytic enzyme using the ClustalX program and ESPript 3.0, and the results are shown in Fig. S4. According to a previous report (52), LaL utilizes conserved Glu19 residues in its active site to exert biological activity, which is similar to the Asp24 of Cly02. Moreover, the saccharide-protein hydrogen bond sites of LaL, including Ala125, Gln68, and Gly38, are also in line with those of Cly02. Although the cut sites of Cly02 remained unclear, we speculated that Cly02 is functionally closely related to LaL. Therefore, the function of Cly02 may involve cleaving the glycan chains between the *N*-acetylmuramic acid and *N*-acetylglucosamine residues of the bacterial PG, similar to that of LaL. Zoocin A is an exoenzyme secreted by a Streptococcus equi subspecies, it exhibits potent antibacterial activity against streptococci, and its solution structure domain participates in target recognition (53). We speculated that the function of Cly03 is similar to that of zoocin A, which prefers to hydrolyze the peptide bond between d-alanine residues in the stem peptide in the PG wall and an l-alanine in the peptide cross bridge (54), causing cell wall breakdown. However, the bactericidal mechanism of Cly01 remained unknown, and we could not find any hydrolase similar to Cly01. However, in accordance with previous reports stating that Cly01 belongs to the NlpC/P60 family of endopeptidases, which hydrolyze the peptide bonds between γ-d-Glu and mDAP (55, 56), we hypothesize that Cly01 may follow this rule as well. Figure 7 summarizes our assumptions and the specific antibacterial mechanism of the Clysins.

## DISCUSSION

Lysins comprise a large structurally and functionally diverse family of bacterium- or phage-encoded PG hydrolases that can destroy bacterial integrity by degrading the chemical bonds of cell wall components (57). As antimicrobial resistance is becoming an increasingly major threat to public health, both novel and effective antibacterial agents are urgently required to bridge the gap formed by traditional antibiotics. Lysins have been considered attractive antibacterial candidates due to their immediate antibacterial effects (24). Since *Enterobacteriaceae*’s lysins are abundant but few studies have focused on Campylobacter-derived lysins. we used “lysin” and other keywords to search in the UniProt Knowledgebase, then we got the protein sequences of 12 lysin candidates. Furthermore, a phylogenetic analysis was carried out on these lysins, of which results confirmed some branches. Cly01, Cly02, and Cly03 were successfully expressed after screening from different branches of lysins, which share a low level of similarity. Further research on the enzyme activity of these three Clysins was executed. Nevertheless, the efficient exterior application of natural lysins is restricted by the OM of Gram-negative bacteria. Therefore, herein, we attempted to fuse three Clysins with TRBA to enhance their exogenous bactericidal activity.

Cloning, expression, and purification were implemented routinely. We successfully obtained seven proteins, including three Clysins (Cly01, Cly02, and Cly03), three TRBA-Clysins (TRBA-Cly01, TRBA-Cly02, and TRBA-Cly03), and TRBA as a negative control. On comparing the results in Fig. 1B and Fig. 2C and D, the fusion of Clysins with TRBA improves the ability of the former to hydrolyze the PG in E. coli BL21. The findings of this study support those of previous studies that Clysins can be combined the TRBA to enhance their antibacterial activity *in vitro* (49, 50). Reportedly, TRBA endows Clysins with the ability to break the OM barrier; however, the mechanisms for the same remain unclarified. Numerous recent studies have been conducted with the aim of expanding the intrinsic ability of lysins to kill Gram-negative pathogens. EDTA can bridge adjacent lipopolysaccharide molecules or competitively displace divalent cations owing to its cationic nature (58), and it has been demonstrated to dramatically improve the bactericidal activity of lysins against Gram-negative bacteria (59–61) However, this effect of EDTA is dependent on specific lysins and bacteria (62, 63). Moreover, the use of EDTA is not fairly suitable for treating systemic infections because of its multiple adverse effects. Cationic amphiphilic peptides (CAPs) are similar to OM-permeabilizing peptides, characterized by a net positive charge that conveniently contrasts with the negative charge of microbial membranes. Thus, they were fused with lysins to enable their transfer across the OM, resulting in PG degradation and Gram-negative bacterial lysis. Briers et al. combined a polycationic nonapeptide (PCNP) with endolysin, which killed several multidrug-resistant strains *in vitro* with a 4- to 5-log reduction within 30 min (39). Similarly, Hu et al. not only verified this type of CAP function but also discovered that artilysin remained highly effective in a mouse infection model (64). We also developed PCNP-Clysins based on previous findings. However, the PCNP-Clysins exhibited poor proteolytic stability and showed no improvement in enzymatic activity (data not shown). Hence, the modification of CAPs is necessary to enable their use in the development of cationic antimicrobial peptide-based lysins.

We hypothesized that colicin K exhibits a potential to assist Clysins to cross the OM and enter the periplasmic space, demolishing the bacterial cell wall of Salmonella spp. via the translocation system in the process. Figure 3B shows that TRBK-Cly02 produced three protein bands in the SDS-PAGE analysis, which may be due to the protein splicing of itself. Although we did not find that TRBK-Clysins affected the survival of Salmonella spp. (data not shown), they showed bactericidal activity against E. coli. Our results revealed that the order of antibacterial activities against E. coli BL21 of the Clysins and six TRB-Clysins was TRBK-Clysins > TRBA-Clysins > Clysins. This suggests that the bactericidal enzymatic activity of the Clysins was further enhanced by inserting TRBK into their N terminus. We propose that TRBK-Cly02 can combat E. coli as a practical antibacterial agent as it was able to considerably reduce E. coli BL21 at a low dose and showed its activity at pH 4 to 8, as reflected in the data mentioned above. The chimeric TRBK-Cly02 designed in our study exhibited more effective anti-E. coli activity at lower doses than previously reported bioengineered lysins, such as colicin-Lysep3 (49), OBPgp279 (39), and Lysep3-D8 (43). This suggests that TRBK-Cly02 might be a preferable bactericidal agent in terms of cost efficiency. Furthermore, we discovered that 32 μg/mL TRBK-Cly02 was sufficient to achieve maximum lytic activity because there was no further decrease in the number of bacteria with increasing concentrations. Hence, by combining the dose-dependent killing curve and time-dependent inhibition curve results (Fig. 4A and C, respectively), we speculate a time- or concentration-dependent action mechanism for the bactericidal effect of lysins as described for the enzyme kinetics. The optimum pH of TRBK-Cly02 was 6, and there was a complete loss of activity at pH values of <3 due to precipitation. We propose two main reasons why TRBK-Cly02 preferred slightly acidic conditions to exhibit antibacterial bioactivity. First, under acidic conditions, the cations allow for the rapid tracking of the OM of Gram-negative bacteria that contains anions (65, 66). Second, each enzyme has an optimum pH at which it exerts maximum enzyme activity, and this pH is determined by the structures and categories of the enzymes. For example, TRBK-Cly02 displayed its optimal bioactivity at pH 6. Notably, this pH-dependent activity trend was similar to those of endolysins LysSE24 (67) and LysF8819.1 (68) but different from those of endolysins Lys394 and LysT144, which were reported to be alkalophilic (67, 69). Although we could not obtain a definitive answer to this question, we have decided to further investigate this pH-dependent regulation in the future to expand the applications of lysins and the accuracy of their enzymatic activity, thereby improving our comprehension regarding the interaction between pathogens and lysins.

In addition to colony-counting and spot-on-lawn assays, directly observing physiological conditions via microscopy can be reasonably helpful to understand the occurrence of events such as cell lysis and aggregation (Fig. 5). It is evident that the E. coli cells were severely damaged, as indicated by their shrunken cell membrane and broken cell wall. Moreover, in TEM observations, the test group exhibited a notably lower bacterial density than the control group (Fig. 5A). This phenomenon may have occurred because numerous cells were lysed into fragments and no specific cell morphology or even bacterial ghosts were visible. Furthermore, fluorescence staining revealed that viable cells were dyed green (in an FITC channel) and evenly distributed in the field of view with intact bacterial morphology (Fig. 5B). However, many red cells were observed following TRBK-Cly02 treatment via a Cy3 filter, and they often aggregated and were irregularly distributed, producing a strong fluorescence intensity. This illustrates that TRBK-Cly02 can cross the OM and damage bacterial cell walls, causing bacterial cell death and aggregation. Cell aggregation may be due to cell lysis or the defense mechanism against environmental stresses, attributed to the increased accumulation of released proteins, polysaccharides, and other molecules at the surface, which provide a metabolically favorable circumstance for bacterial cells and serve as nutritional signals to trigger biofilm formation (70, 71). The cell aggregation phenomenon also demonstrated the bactericidal effect of TRBK-Cly02 from another perspective.

Herein, the colicins used had a two-domain structure comprising an N-terminal T domain responsible for transporting the lysins across the OM and a central RB domain responsible for binding the colicins to the OM cell receptor. Colicins A and K were classified as A-group colicins based on the OM receptors they bind and the translocation mechanism through the host periplasm they rely on, and these colicins are imported through the Tol-dependent translocation system. Colicins A and K bind to the BtuB and Tsx receptors to enter the target cells. Thereafter, OmpF porin acts as a channel to allow the unfolded T domain to cross the OM and come in contact with TolA, thereby activating the Tol-dependent translocation system (45, 72, 73) (Fig. 7). The receptors bound by colicins could be restricted to bacterial species and strains; therefore, these bioengineered lysins, also known as lysocins, only display application potential on a narrow spectrum of bacterial species. This is supported by the incompetence of TRBA-Clysins and TRBK-Cly02 against Salmonella *in vitro* (Fig. 6; Fig. S1). Consequently, from the bioengineering perspective, novel lysins exhibit a somewhat multifactorial coregulation bactericidal bioactivity, such as the activity of native lysins, preferred translocation system colicins, and the prevalent translocation system in bacterial metabolism (28, 50, 52). Notably, Cly01, Cly02, and Cly03, which are secreted by Campylobacter, exhibit bacteriostatic activity against E. coli; thus, selecting lysins to kill pathogens is not necessarily limited to specific sources. This is consistent with previous findings (53, 54). Presumably, each engineered portion of lysins affects their antibacterial activity, and further empirical research is warranted to determine the optimum combination mode for targeting a specific pathogen (55, 56).

Lysins have tremendous therapeutic potential compared to conventional antimicrobials due to their diversity and targeted mode of action. Because engineered lysins are about to enter clinical use, some studies have been carried out on whether bacteria will develop resistance to lysins; these results could serve as evidence that similar to native phage endolysins (74–76), engineered phage endolysins have a low probability of developing resistance *in vitro* (77). For instance, chimeric antistaphylococcal endolysin ClyS, which combines two endolysins derived from the Twort phage and phiNM3 phage, which did not induce resistance since MIC_90_ values for ClyS against two S. aureus strains remain the same after an 8-day exposure in the presence of increasing concentrations. In contrast, the MIC_90_ values for mupirocin obviously increased (78). Additionally, autolysins have not been reported to potentially impact bacterial evolution, possibly because of highly conserved PG structures (23, 24). However, resistance to lysozymes can be induced *in vitro* even in highly susceptible species, such as Clostridioides difficile (79) and Streptococcus suis (80). The mechanisms of bacterial resistance to lysozymes include peptidoglycan modifications (81) and the production of inhibitor proteins (82), which can possibly endow bacteria with the ability to resist lysins in the future. Although there are currently a handful of lysin-based products on the market (40, 83, 84), some roadblocks can be encountered during the development of lysins as treatment methods in a clinical setting. On the one hand, the main challenge is drug delivery methods since lysins are immediately vulnerable to enzymes, different pH levels, and immune cells in the human body (85, 86). On the other hand, the process of obtaining approval from the regulatory body is tedious, consisting of evaluations of the manufacture, clinical trials, and safety (87), which means that the success rate of lysins with viable medical application is considerably low. However, the development of veterinary-based lysin products may be faster since the restrictions outlined for animal use allow lysins to be used for the treatment of several diseases (88, 89). Moreover, it is worth mentioning that lysins have been shown to be effective in eliminating biofilms formed by pathogens on different surfaces in the food industry (90), and various lysins have been demonstrated to be good candidates for use in food against bacteria (91). Although there are many positive uses of lysins in ordinary clinical therapy, animal treatment and food safety, lysin usage is still in its infancy. Hence, researchers need to focus on the modifications of lysins in the future, which allow them to maintain high activity while possessing other desirable traits, depending on the circumstances of their use, and even develop novel commercial treatment products.

In conclusion, we validated the antibacterial activity of three Clysins for the first time and devised six bioengineered TRB-Clysins by fusing the TRB domains of colicins with native lysins. This demonstrated the advantages of the colicin fusion method, which allowed the extrinsically applied lysins to overcome the OM barrier of Gram-negative bacteria. In particular, this study offers a new method of combining colicin K with lysins to kill E. coli. Furthermore, TRBK-Cly02 demonstrated efficient bactericidal activity against E. coli in the pH range of 4 to 8 even at a low dose. Therefore, TRBK-Cly02 could be used as an alternative therapeutic agent to combat E. coli with low bacterial resistance.

## MATERIALS AND METHODS

### Bacteria and growth conditions.

The bacterial strains used in this study are listed in Table S4 in the supplemental material. E. coli and Salmonella strains were cultured on LB medium or LB agar plates (Coolaber, China) at 37°C. C. jejuni strains were generally grown in brain heart infusion broth or on brain heart infusion agar plates (Oxoid, United Kingom) at 42°C under microaerophilic conditions with ~5% O_2_, 10% CO_2_, and 85% N_2_.

### Protein cloning, expression, and purification.

The TRB domains of colicin A and colicin K were analyzed using Simple Modular Architecture Research Tool (http://smart.embl-heidelberg.de). The sequence published by the NCBI was used for codon optimization and His_6_ tag addition to the original DNA sequence of Cly01 (GenBank accession no. FAVE01000001.1), Cly02 (GenBank accession no. ANNH01000031.1), Cly03 (GenBank accession no. AANOQR020000014.1), TRBA (GenBank accession no. M37402.1), and TRBK (GenBank accession no. ABBXIW010000017.1). In particular, a signal peptide in Cly01 was removed. A linker (GGGGS) was used for individually combining TRBA-Cly01, TRBA-Cly02, TRBA-Cly03, TRBK-Cly01, TRBK-Cly02, and TRBK-Cly03. The codon-optimized DNA sequences of the lysins were then synthesized by General Biol Co., Ltd. (Anhui, China). All of the amino acid and DNA sequences used in this study are listed in Table S1 and Table S2, respectively.

All genes were amplified using primers shown in Table S3, and restriction sites were added on each end of the primers. Afterward, these target gene products and the pET-28a(+) vector were digested with the corresponding restriction enzymes (TaKaRa, Japan). Thereafter, the linearized fragments were combined with T4 DNA ligase (TaKaRa, Japan) to generate recombinant plasmids, such as pET-Cly01, pET-Cly02, pET-Cly03, pET-TRBA-Cly01, pET-TRBA-Cly02, pET-TRBA-Cly03, pET-TRBA, pET-TRBK-Cly01, pET-TRBK-Cly02, pET-TRBK-Cly03, and pET-TRBK. These plasmids were transformed into E. coli DH5α for cloning. Following PCR detection and sequence confirmation, the correct recombinant plasmids were transformed into E. coli BL21(DE3) for protein expression.

E. coli BL21(DE3) was used to express Cly01, Cly02, Cly03, TRBA, TRBA-Cly01, TRBA-Cly02, TRBA-Cly03, TRBK, TRBK-Cly01, TRBK-Cly02, and TRBK-Cly03 at 16°C with overnight shaking at 200 rpm in LB medium containing 30 μg/mL kanamycin. Protein expression was induced at the log phase (OD_600_ of 0.8) with 0.1 mM IPTG (isopropyl-β-d-1-thiogalactopyranoside). The cells were harvested by centrifugation at 6,000 × *g* for 10 min. Thereafter, they were washed twice with 10 mM PBS and resuspended in 50 mM lysis buffer without imidazole (NaH_2_PO_4_, 300 mM NaCl [pH 8]). The pellets were sonicated on ice at a frequency of 20 kHz using an ultrasonicator (JY88-IIN, China) for 30 min (5 s off, 3 s on), and the supernatant was collected after centrifugation of the pellets at 8,000 × *g* for 20 min at 4°C. Immobilized metal ion affinity chromatography was used to purify the proteins. Before loading, Ni-Sepharose (GE Healthcare, USA) was equilibrated with 10 column volumes of cold lysis buffer (50 mM NaH_2_PO_4_, 300 mM NaCl, 10 mM imidazole [pH 8]). The proteins were fully combined with Ni-Sepharose, and then the column was washed with wash buffer (50 mM NaH_2_PO_4_, 300 mM NaCl, 20 mM imidazole [pH 8.0]). The proteins were then eluted with the same buffer comprising 250 mM imidazole. The proteins containing 6×His tags were collected and dialyzed against PBS (10 mM Na_2_HPO_4_, 2 mM KH_2_PO_4_, 137 mM NaCl, 2.7 mM KCl, and pH 7.4) overnight at 4°C. The proteins’ purity and amount were assessed using SDS-PAGE and a bicinchoninic acid (BCA) protein assay kit (Beyotime Biotechnology, China), respectively. All proteins were filter sterilized through 0.22-μm-pore filter units (Merck KGaA, Darmstadt, Germany) and stored in 20% glycerol at −20°C for subsequent experiments.

### Antibacterial activity test.

A previously reported plate culture count method (49) was used with slight modifications to assess the sterilization activities of the native and bioengineered Clysins. In brief, E. coli BL21 was inoculated in LB broth at 37°C with shaking at 200 rpm for 4 h. Thereafter, the cells in the log phase were centrifuged at 5,000 × *g* for 1 min and resuspended with sterile PBS to a final density of 2 × 10^8^ to ~8 × 10^8^ CFU/mL. Furthermore, 90 μL bacterial inoculum was added to a 10-μL dilution of protein and PBS, making the final concentration 1 μM (24.8 μg/mL for Cly01, 25.6 μg/mL for Cly02, 32.2 μg/mL for Cly03, 64.9 μg/mL for TRBA-Cly01, 65.8 μg/mL for TRBA-Cly02, 72.6 μg/mL for TRBA-Cly03, 59.3 μg/mL for TRBK-Cly01, 60.1 μg/mL for TRBK-Cly02, 66.8 μg/mL for TRBK-Cly03, 40.0 μg/mL for TRBA, and 34.3 μg/mL for TRBK). Ten microliters of buffer without proteins was mixed with 90 μL bacterial inoculum as a negative control. All mixtures were incubated without shaking at 37°C for 16 h, serially diluted 10-fold, and plated on LB agar plates, which were incubated overnight at 37°C to determine the CFU. The antibacterial activity was quantified as the relative inactivation level in log units [log_10_(*N*_0_/*N_i_*), where *N*_0_ is the number of untreated cells and *N_i_* is the number of residual cells counted after treatment].

For the plate lysis assay, the E. coli BL21 culture in the log phase was diluted 1,000-fold with 0.5% soft LB agar (50°C) and subsequently pooled to the top of the LB agar plates. After the plate completely solidified, 5 μL of each diluted sample containing 0.1 nmol protein (2.48 μg for Cly01, 2.56 μg for Cly02, 3.21 μg for Cly03, 6.50 μg for TRBA-Cly01, 6.58 μg for TRBA-Cly02, 7.26 μg for TRBA-Cly03, 5.93 μg for TRBK-Cly01, 6.01 μg for TRBK-Cly02, 6.68 μg for TRBK-Cly03, 4.00 μg for TRBA, and 3.43 μg for TRBK) was spotted onto specified areas of the double-layer plate and cultivated overnight at 37°C. Buffer without lysins was spotted as a negative control. The diameters of the inhibition zone were measured to evaluate the capacity of the lysing germ.

### MIC assay.

The MIC of TRBK-Cly02 against E. coli BL21 was determined according to the Clinical and Laboratory Standards Institute, with some modifications. Briefly, TRBK-Cly02 was prepared in Mueller-Hinton (MH) broth (Oxoid, United Kingdom) and serially diluted 2-fold in 96-well plates to achieve final concentrations of 0.65 to 168 μg/mL. Pure MH broth with and without bacteria was used as the positive and negative controls, respectively. Afterward, test strains in the exponential growth phase (10^8^ CFU/mL) were added to each well, followed by incubation at 37°C for 18 h. Finally, the MIC was defined as the minimum concentration that completely inhibited bacterial growth.

### Evaluation of the antibacterial performance of TRBK-Cly02.

Different TRBK-Cly02 doses (0, 2, 4, 8, 16, 32, 64, 128, and 256 μg/mL) were added to the bacterial suspension (pH 7.4) to demonstrate the correlation between concentration and bactericidal bioactivity. Thereafter, the antibacterial activity was assessed as previously mentioned (see the “Antibacterial activity test” section). Finally, the concentration-kill curve was illuminated using GraphPad Prism8.

To clarify the regular pattern of the external factors affecting the sterilization bioactivity of TRBK-Cly02, antibacterial activity tests were performed as described in a previous study (50), with some modifications. The effects of pH were analyzed using the E. coli BL21 strain cultured to the logarithmic growth stage. The cells were washed and resuspended with an equal volume of PBS at different pH values (pH 3 to 8). Thereafter, the final MIC and 5× MIC of TRBK-Cly02 were added into the bacterial liquid with different pH values. The antibacterial activity was evaluated as mentioned before (see the “Antibacterial activity test” section).

First, the E. coli BL21 strain was incubated on a shaker until the OD_600_ was 0.2 to illustrate the relationship between time and bactericidal bioactivity. Second, 32 μg/mL TRBK-Cly02 or an equal volume of the buffer without proteins was incubated with the bacterial culture at 37°C with shaking at 200 rpm. Afterward, the OD_600_ was measured every 2 h using a UV spectrophotometer (UV-6100S, China). The monitoring of the bacterial growth was stopped after 12 h. Finally, the time-kill curve was illuminated using GraphPad Prism 8.

### TEM and fluorescence microscopy observations.

For the TEM analysis, 1 mL E. coli BL21 in the log phase was washed and resuspended with 1 mL PBS before being incubated with 32 μg/mL of TRBK-Cly02 or sterile PBS buffer without proteins (as the negative control) at 37°C for 16 h. The cells were fixed with 2.5% glutaraldehyde and 2% osmic acid. Furthermore, the cells were washed with anhydrous ethanol several times at 24°C for dehydration. The samples were embedded and polymerized and were subsequently sliced to 70- to 100-nm-thick sections using an ultramicrotome (Leica, Germany). Thereafter, the samples were deposited on carbon-coated copper grids. Finally, the morphology of the bacterial cells was observed via TEM (Talos L120C; Thermo Scientific, Pardubice, Czech Republic).

For the fluorescence microscopy analysis, the E. coli BL21 cells were prepared using the same method described above. Bacterial staining was performed according to the live and dead bacterial staining kit instructions (Yeasen Biotech, China). Briefly, 100 μL of the bacterial mixture was washed and resuspended in 0.85% NaCl then added to a 1 μL mixture of DMAO and EthD-III (1:1). Thereafter the samples incubated in the dark at 24°C for 15 min. The labeled cells were visualized using an inverted microscope system (Eclipse Ti2, Japan). Live cells were stained with green DMAO dye, whereas dead cells were stained with red EthD-III dye.

### Antimicrobial spectrum of TRBK-Cly02.

The 11 strains (the origins of the bacteria are listed in Table S4) were inoculated in broth under optimum conditions until they reached the log phase. Then the bacterial concentrations were adjusted to a final density of 2 × 10^8^ to ~8 × 10^8^ CFU/mL, and the antibacterial activity test was performed as mentioned above. The final colonies were counted after being washed and resuspended in PBS (pH 6) containing 5 mM EDTA and incubated overnight with TRBK-Cly02 under the concentration of 60.1 μg/mL at 37°C for 16 h.

### Sequence analysis.

To compare Clysins with other known lysins, the Clysin’s protein sequence was tested against the GenBank database of proteins using blastp. Alignment of Cly02 and three similar lysins’ amino acid sequences was conducted with the ClustalX program. Visualization of the alignment and annotation of secondary structure elements were performed using ESPript 3.0 (92) (http://espript.ibcp.fr/ESPript/cgi-bin/ESPript.cgi).

### Statistical methods.

Data are presented as the mean ± standard deviation, and *n* = 3 for each group from technical triplicate experiments. Statistical analyses were executed using IBM SPSS Statistics (version 26.0). For the data in Fig. 1B, Fig. 2C, and Fig. 3C, the log_10_ reductions of cells were analyzed by one-way analysis of variance (ANOVA) followed by Tukey’s multiple-comparison test (95% confidence interval). For the data in Fig. 4C, the OD_600_ values at each time point between two experimental groups were analyzed by the paired-sample *t* test. *P* values of <0.05 were considered statistically significant.

### Data availability.

Data obtained in this study were deposited into NCBI under BioProject accession no. PRJNA926004.
