# Supplementary material for: Identification of Three Campylobacter Lysins and Enhancement of Their Anti-Escherichia coli Efficacy Using Colicin-Based Translocation and Receptor-Binding Domain Fusion
Source: Microbiol Spectr. 2023 Feb 7;11(2):e04515-22. doi: 10.1128/spectrum.04515-22 (PMC10100823; doi:10.1128/spectrum.04515-22)
Supplement: Supplemental file 1 — Tables S1 to S4 and Fig. S1 to S4. Download spectrum.04515-22-s0001.pdf, PDF file, 1.1 MB [file spectrum.04515-22-s0001.pdf]

Supplemental material

Identification of three *Campylobacter* lysins and enhancement of their anti-*Escherichia coli* efficiency using colicin-based translocation and receptor-binding domains fusion

Authors: Peiqi Liu[a#], Xinying Dong[a#], Xuewei Cao[a], Qianmei Xie[a], Xiuqin Huang[a], Jinfei Jiang[a], Huilin Dai[a], Zheng Tang[a], Yizhen Lin[a], Saixiang Feng[a]*, Kaijian Luo[a]*

[a]College of Veterinary Medicine, South China Agricultural University, Guangzhou, China.

***Correspondence:**

Saixiang Feng: Tel/Fax, +86-20-85280718; E-mail: fengsx@scau.edu.cn

Kaijian Luo: Tel/Fax, +86-20-85280240; E-mail: kjluo@scau.edu.cn

[#]Peiqi Liu and Xinying Dong contributed equally to this article.

Content

Table S1. The original amino acid sequences used as templates.

Table S2. DNA sequences used in this study.

Table S3. Primers used in this study.

Table S4. Strains and plasmids used in this study.

Fig. S1. Antibacterial activity test of Clysins and TRBA-Clysins against *Salmonella* spp.

Fig. S2. Effects of pH on the activity of Cly02 against *E. coli* BL21 in PBS.

Fig. S3. Antimicrobial spectrum of Cly02 and TRBK-Cly02 against *E. coli*, *Salmonella* spp., and *Campylobacter jejuni* strains.

Fig. S4. Sequence analysis of Cly02 with lytic transglycosylase, lambda lysozyme, and *Escherichiavirus* T4 endolysin.

Table S1. The original amino acid sequences used as templates.

Name	Amino Acid Sequences	Accession Code
CJ01	MWFKGVLTISALLLILSGCSTKSIDPYGEQNSYDNIGSKYQKNDT<br>TSGYNFNYNQAADEFYNINLSKYLNKKLGNDCSGFVSLVNEDS<br>KSLYFDENIVNNFYDKNGRKSQAIFNLYKSQNKISYTDPKPGDL<br>VFFNNTTSRTNKSKNKAITHLGIIDKVESNGTITFVHNINGKNIKS<br>VMNLKHKNTHKLNGKKINAYIILKCQTPVCLISNKFAGFGKVDK<br>FVQID	CUU71468.1
CJ02	MNNFINSFNESKSITRVDKGMWVDGDKGVGVKADGDIRILRIR<br>AFMCAIKYGEGTSGNNGYEINVGGKLFTKDYGKDFSDHPRYYV<br>KSLDSSAAGAYQIKSSTWDMILKKYKKTYDITDFSPANQDKACL<br>VLIKHIRNALDLIVDEKIDEAVRSRTDNDKKRLHYEWASMPDSP<br>YGQRTITMEKFMEYYMHHLELEEIGISNLAISNKDIKKFLN	ERJ25909.1
CJ03	MNTDALINWFESRRGKLTYSMYGSRNGSDGTADCSGSVSQALK<br>EAGIPIKGLPSTVTLGTQLAQNGFIRVSRNTDWTAQRGDIVLMS<br>WGADMSTSGGAGGHVGVMEDANTFISVDYWTGGAAGQAVSS<br>HNWDQYYNAQRPRYIEAWRYNGAGGNQQPNTSVSQTPTRKPD<br>SKAYYLANDVAFVNGIYQIKCDYLAPVGFDWNDNGVPVGLVN<br>WVDENGNNVRDGADKDFKPGMYFSFEIDEAHIADTGEGGYYG<br>GYYWRKFEFGQFGTVWLSCRDKDDLVNYYK	NHG91284.1
colicin A	MPGFNYGGKGDGTGWSSERGSGPEPGGGSHGNSGGHDRGDSS<br>NVGNESVTVMKPGDSYNTPWGKVIINAAGQPTMNGTVMTADN<br>SSMVPYGRGFTRVLNSLVNNPVSPAGQNGGKSPVQTAVENYLM<br>VQSGNLPPGYWLSNGKVMTEVREERTSGGGGKNGNERTWTVK<br>VPREVPQLTASYNEGMRIRQEAADRARAEANARALAEEEARAI<br>ASGKSKAEFDAGKRVEAAQAAINTAQLNVNNLSGAVSAANQVI<br>TQKQAEMTPLKNELAAANQRVQETLKFINDPIRSRIHFNMRSGLI<br>RAQHNVDTKQNEINAAVANRDALNSQLSQANNILQNARNEKSA<br>ADAALSAATAQRLQAEAALRAAAEAAEKARQRQAEEAERQRQ<br>AMEVAEKAKDERELLEKTSELIAGMGDKIGEHLGDKYKAIAKD<br>IADNIKNFQGKTIRSFDDAMASLNKITANPAMKINKADRDALVN	WP_008323639.1

	AWKHVDAQDMANKLGNLSKAFKVADVVMKVEKVREKSIEGY	
	ETGNWGPLMLEVESWVLSGIASSVALGIFSATLGAYALSLGVPA	
	IAVGIAGILLAAVVGALIDDKFADALNNEIIRPAH	
colicin K	MAKELSGYGPTAGESMGGTGANLNQQGGNNNSNSGVHWGGG	EHY5305768.1
	SGHGNNGGQGNSNSSGSTSTVMKTGESYLTPWGDVVINNDGLP	
	VMNGIVMTEENSTLVDNPFGGVSRVLNSLISDMPSLFAESSGNN	
	NNNTASVNTAPTNAQVSDMDKSSKVVSNVINEKQKQKNKIATQ	
	ISEKQKKIEEMKKVFKHHSYHGITDLERDVDELQKKSNQLDADI	
	SKLNSYKNTLQSKIGDVNKQKEAEEKARENAEVAEHETLNEEK	
	QAVAEAEKRLAEAKAELAKAESDVQSKQATVSRVAGELENAQ	
	KSVDVKVTGFPGWRDVQKKLQRQLEAKQAEYSAVENELKNAV	
	SFRDGKAAEVKEAEQKLKEAQDALEKSQIKDAVDTMVGFYQYI	
	TEQYGEKYAKIAQDLAEKSKGKKIQGVDEALAAFEKYKNVLDK	
	KFSKVDRDAIFNALESVNYDELSKNLTKISKSLKITSRVSFLYDV	
	GSDFKNAIETGNWRPLFVTLEKSAVDVGVAKIVALMFSFIVGVP	
	LGFWGIAIVTGIV SSYIGDDELSKLNELLGI	

For CJ01, the underlined region is a signal peptide; for colicin A and colicin K the underlined regions belong to the C-terminal cytotoxic domains. The underlined regions were removed when synthesize DNA sequences.

Table S2. DNA sequences used in this study.

Name	DNA Sequence (5′ to 3′)
Cly01	ATGTGCTCAACAAAATCTATTGATCCTTACGGTGAGCAGAATAGTTATGATAATAT
	TGGGTCAAAATACCAGAAAAATGATACGACCTCAGGTTACAATTTCAATTATAACC
	AGGCGGCTGACGAATTCTACAATATTAATCTGAGCAAGTACCTTAATAAGAAACTC
	GGGAACGATTGCTCGGGGTTTGTAAGCTTAGTTAACGAAGACTCCAAGAGTCTGTA
	TTTCGATGAGAACATTGTCAATAATTTTTACGACAAGAACGGTCGCAAGTCCCAAG
	CTATCTTCAACCTTTATAAGTCCCAAAACAAAATTAGCTATACCGACCCTAAACCG
	GGCGATTTAGTATTTTTTAACAATACCACAAGCCGGACGAACAAATCTAAAAATAA
	GGCAATTACTCACCTCGGTATTATCGACAAAGTTGAGTCTAATGGTACTATCACGTT
	CGTTCACAACATTAACGGGAAGAATATCAAAAGCGTAATGAACTTGAAGCACAAG
	AACACACATAAATTAAATGGCAAGAAAATCAACGCTTATATTATTCTCAAGTGTCA
	AACGCCTGTATGTTTGATTTCCAACAAATTCGCTGGCTTTGGGAAAGTGGACAAAT
	TTGTGCAAATTGATGGTTCGCATCACCACCACCACCATTAA
Cly02	ATGAACAACTTTATTAACTCTTTTAACGAATCCAAATCTATCACACGGGTGGATAA
	AGGCATGTGGGTCGATGGTGACAAAGGTGTTGGGGTGAAGGCCGATGGCGACATC
	CGTATTCTGCGTATTCGGGCGTTCATGTGCGCAATCAAATATGGGGAGGGCACTAG
	CGGCAACAACGGTTACGAAATCAACGTGGGCGGGAAATTATTTACCAAAGACTAT
	GGTAAGGATTTTTCTGATCACCCACGGTATTACGTCAAAAGTCTCGACTCCTCAGCA
	GCTGGTGCATACCAGATTAAAAGTTCCACATGGGACATGATTCTGAAGAAGTATAA
	GAAAACTTACGATATTACCGATTTCTCACCAGCAAACCAGGATAAGGCTTGCCTTG
	TGCTGATTAAACACATTCGTAATGCACTTGACCTGATCGTGGACGAGAAGATTGAT
	GAAGCCGTCCGGTCTCGTACCGATAATGATAAGAAACGCCTGCACTACGAATGGGC
	GTCAATGCCAGACTCTCCATATGGGCAACGTACTATTACTATGGAGAAGTTTATGG
	AATACTACATGCATCATTTGGAGCTCGAAGAGATTGGTATCTCGAACCTGGCCATC
	TCCAATAAAGACATCAAAAAATTCCTCAATGGTTCGCATCATCATCACCATCATTA
	A
Cly03	ATGAATACGGACGCCTTGATTAACTGGTTTGAGAGCCGTCGCGGCAAACTCACTTA
	TTCGATGTACGGGTCGCGTAATGGTTCTGATGGGACAGCAGATTGCAGCGGGTCGG
	TCAGTCAAGCGTTAAAGGAGGCGGGGATTCCTATTAAAGGCCTTCCATCCACGGTT
	ACTCTGGGCACCCAGCTCGCCCAGAATGGCTTCATTCGGGTGTCACGGAATACTGA

	CTGGACCGCTCAGCGGGGCGACATCGTGCTTATGAGCTGGGGGGCTGATATGAGTA
	CGTCCGGTGGCGCCGGTGGCCATGTGGGGGTAATGGAAGACGCTAACACTTTTATC
	AGCGTTGACTATTGGACGGGTGGTGCGGCTGGTCAGGCTGTATCTTCCCACAATTG
	GGACCAGTATTACAACGCTCAACGTCCTCGCTATATCGAAGCATGGCGCTATAATG
	GCGCCGGGGGGAATCAACAGCCTAACACCTCCGTTAGCCAAACGCCTACTCGTAAA
	CCAGATTCTAAAGCATACTATCTTGCTAATGACGTTGCTTTTGTTAACGGGATCTAC
	CAGATTAAGTGCGATTACCTTGCCCCGGTTGGTTTTGACTGGAACGATAATGGTGT
	GCCGGTTGGGCTGGTGAACTGGGTCGACGAGAACGGCAATAATGTACGTGATGGC
	GCGGACAAGGACTTTAAACCAGGCATGTACTTCTCCTTTGAGATTGACGAGGCCCA
	TATCGCCGATACAGGGGAGGGCGGCTATTATGGCGGGTATTACTGGCGCAAATTTG
	AATTCGGTCAATTCGGGACTGTGTGGCTTTCCTGTCGTGACAAGGATGATCTCGTAA
	ACTACTACAAAGGTTCGCATCATCATCACCATCATTAA
TRBA	CCTGGATTTAATTATGGTGGAAAAGGTGATGGAACCGGCTGGAGCTCAGAACGTGG
	GAGTGGTCCAGAGCCGGGTGGTGGTAGCCATGGAAATAGTGGTGGGCACGATCGT
	GGAGATTCTTCCAACGTAGGTAATGAGTCTGTGACGGTAATGAAACCAGGGGATTC
	GTATAACACCCCGTGGGGAAAAGTCATCATCAATGCTGCAGGCCAGCCGACCATGA
	ACGGAACGGTGATGACCGCTGATAATTCATCGATGGTTCCTTACGGCAGAGGGTTT
	ACACGGGTTTTAAATTCCCTGGTCAATAATCCTGTTTCGCCGGCAGGTCAGAATGG
	CGGGAAGTCTCCTGTTCAGACTGCTGTGGAAAATTATCTGATGGTACAGTCAGGAA
	ACCTGCCACCGGGCTACTGGCTCAGTAATGGCAAGGTTATGACGGAGGTTCGTGAG
	GAACGTACTTCTGGCGGCGGTGGGAAAAACGGGAACGAGCGAACCTGGACTGTGA
	AAGTTCCCCGGGAAGTACCTCAGCTTACGGCATCCTATAACGAGGGGATGAGAATC
	CGACAGGAGGCAGCTGACCGTGCCAGAGCGGAAGCAAATGCCCGCGCTCTGGCTG
	AAGAGGAAGCCCGTGCCATCGCATCAGGAAAGAGCAAAGCTGAGTTTGATGCAGG
	TAAGCGGGTGGAGGCCGCACAGGCAGCGATTAATACAGCACAACTCAATGTTAAT
	AACCTCAGCGGCGCTGTCAGTGCTGCAAATCAGGTTATAACTCAGAAACAGGCTGA
	AATGACGCCCCTGAAAAATGAGCTTGCAGCCGCTAACCAGCGTGTCCAGGAGACG
	CTTAAATTTATCAATGATCCTATTCGTAGCCGGATTCATTTTAATATGCGAAGTGGC
	CTGATTCGCGCTCAACATAACGTTGATACTAAACAGAATGAAATTAATGCAGCAGT
	GGCTAACCGTGATGCTCTGAATAGCCAATTGTCTCAGGCTAATAATATCCTGCAGA
	ATGCCCGGAACGAAAAGAGTGCGGCTGATGCAGCACTTTCAGCTGCCACAGCACA
	GCGGTTACAGGCAGAAGCCGCACTCAGGGCTGCTGCTGAGGCTGCAGAAAAAGCG
	CGCCAGCGCCAGGCTGAAGGTTCGCATCATCATCACCATCATTAA

TRBA-Cly01	CCTGGATTTAATTATGGTGGAAAAGGTGATGGAACCGGCTGGAGCTCAGAACGTGG<br>GAGTGGTCCAGAGCCGGGTGGTGGTAGCCATGGAAATAGTGGTGGGCACGATCGT<br>GGAGATTCTTCCAACGTAGGTAATGAGTCTGTGACGGTAATGAAACCAGGGGATTC<br>GTATAACACCCCGTGGGGAAAAGTCATCATCAATGCTGCAGGCCAGCCGACCATGA<br>ACGGAACGGTGATGACCGCTGATAATTCATCGATGGTTCCTTACGGCAGAGGGTTT<br>ACACGGGTTTTAAATTCCCTGGTCAATAATCCTGTTTCGCCGGCAGGTCAGAATGG<br>CGGGAAGTCTCCTGTTCAGACTGCTGTGGAAAATTATCTGATGGTACAGTCAGGAA<br>ACCTGCCACCGGGCTACTGGCTCAGTAATGGCAAGGTTATGACGGAGGTTCGTGAG<br>GAACGTACTTCTGGCGGCGGTGGGAAAAACGGGAACGAGCGAACCTGGACTGTGA<br>AAGTTCCCCGGGAAGTACCTCAGCTTACGGCATCCTATAACGAGGGGATGAGAATC<br>CGACAGGAGGCAGCTGACCGTGCCAGAGCGGAAGCAAATGCCCGCGCTCTGGCTG<br>AAGAGGAAGCCCGTGCCATCGCATCAGGAAAGAGCAAAGCTGAGTTTGATGCAGG<br>TAAGCGGGTGGAGGCCGCACAGGCAGCGATTAATACAGCACAACTCAATGTTAAT<br>AACCTCAGCGGCGCTGTCAGTGCTGCAAATCAGGTTATAACTCAGAAACAGGCTGA<br>AATGACGCCCCTGAAAAATGAGCTTGCAGCCGCTAACCAGCGTGTCCAGGAGACG<br>CTTAAATTTATCAATGATCCTATTCGTAGCCGGATTCATTTTAATATGCGAAGTGGC<br>CTGATTCGCGCTCAACATAACGTTGATACTAAACAGAATGAAATTAATGCAGCAGT<br>GGCTAACCGTGATGCTCTGAATAGCCAATTGTCTCAGGCTAATAATATCCTGCAGA<br>ATGCCCGGAACGAAAAGAGTGCGGCTGATGCAGCACTTTCAGCTGCCACAGCACA<br>GCGGTTACAGGCAGAAGCCGCACTCAGGGCTGCTGCTGAGGCTGCAGAAAAAGCG<br>CGCCAGCGCCAGGCTGAAGGTGGAGGAGGATCCATGTGCTCAACAAAATCTATTG<br>ATCCTTACGGTGAGCAGAATAGTTATGATAATATTGGGTCAAAATACCAGAAAAAT<br>GATACGACCTCAGGTTACAATTTCAATTATAACCAGGCGGCTGACGAATTCTACAA<br>TATTAATCTGAGCAAGTACCTTAATAAGAAACTCGGGAACGATTGCTCGGGGTTTG<br>TAAGCTTAGTTAACGAAGACTCCAAGAGTCTGTATTTCGATGAGAACATTGTCAAT<br>AATTTTTACGACAAGAACGGTCGCAAGTCCCAAGCTATCTTCAACCTTTATAAGTCC<br>CAAAACAAAATTAGCTATACCGACCCTAAACCGGGCGATTTAGTATTTTTTAACAA<br>TACCACAAGCCGGACGAACAAATCTAAAAATAAGGCAATTACTCACCTCGGTATTA<br>TCGACAAAGTTGAGTCTAATGGTACTATCACGTTCGTTCACAACATTAACGGGAAG<br>AATATCAAAAGCGTAATGAACTTGAAGCACAAGAACACACATAAATTAAATGGCA<br>AGAAAATCAACGCTTATATTATTCTCAAGTGTCAAACGCCTGTATGTTTGATTTCCA<br>ACAAATTCGCTGGCTTTGGGAAAGTGGACAAATTTGTGCAAATTGATGGTTCGCAT<br>CACCACCACCACCATTAA

TRBA-Cly02	CCTGGATTTAATTATGGTGGAAAAGGTGATGGAACCGGCTGGAGCTCAGAACGTGG<br>GAGTGGTCCAGAGCCGGGTGGTGGTAGCCATGGAAATAGTGGTGGGCACGATCGT<br>GGAGATTCTTCCAACGTAGGTAATGAGTCTGTGACGGTAATGAAACCAGGGGATTC<br>GTATAACACCCCGTGGGGAAAAGTCATCATCAATGCTGCAGGCCAGCCGACCATGA<br>ACGGAACGGTGATGACCGCTGATAATTCATCGATGGTTCCTTACGGCAGAGGGTTT<br>ACACGGGTTTTAAATTCCCTGGTCAATAATCCTGTTTCGCCGGCAGGTCAGAATGG<br>CGGGAAGTCTCCTGTTCAGACTGCTGTGGAAAATTATCTGATGGTACAGTCAGGAA<br>ACCTGCCACCGGGCTACTGGCTCAGTAATGGCAAGGTTATGACGGAGGTTCGTGAG<br>GAACGTACTTCTGGCGGCGGTGGGAAAAACGGGAACGAGCGAACCTGGACTGTGA<br>AAGTTCCCCGGGAAGTACCTCAGCTTACGGCATCCTATAACGAGGGGATGAGAATC<br>CGACAGGAGGCAGCTGACCGTGCCAGAGCGGAAGCAAATGCCCGCGCTCTGGCTG<br>AAGAGGAAGCCCGTGCCATCGCATCAGGAAAGAGCAAAGCTGAGTTTGATGCAGG<br>TAAGCGGGTGGAGGCCGCACAGGCAGCGATTAATACAGCACAACTCAATGTTAAT<br>AACCTCAGCGGCGCTGTCAGTGCTGCAAATCAGGTTATAACTCAGAAACAGGCTGA<br>AATGACGCCCCTGAAAAATGAGCTTGCAGCCGCTAACCAGCGTGTCCAGGAGACG<br>CTTAAATTTATCAATGATCCTATTCGTAGCCGGATTCATTTTAATATGCGAAGTGGC<br>CTGATTCGCGCTCAACATAACGTTGATACTAAACAGAATGAAATTAATGCAGCAGT<br>GGCTAACCGTGATGCTCTGAATAGCCAATTGTCTCAGGCTAATAATATCCTGCAGA<br>ATGCCCGGAACGAAAAGAGTGCGGCTGATGCAGCACTTTCAGCTGCCACAGCACA<br>GCGGTTACAGGCAGAAGCCGCACTCAGGGCTGCTGCTGAGGCTGCAGAAAAAGCG<br>CGCCAGCGCCAGGCTGAAGGTGGAGGAGGATCCATGAACAACTTTATTAACTCTTT<br>TAACGAATCCAAATCTATCACACGGGTGGATAAAGGCATGTGGGTCGATGGTGACA<br>AAGGTGTTGGGGTGAAGGCCGATGGCGACATCCGTATTCTGCGTATTCGGGCGTTC<br>ATGTGCGCAATCAAATATGGGGAGGGCACTAGCGGCAACAACGGTTACGAAATCA<br>ACGTGGGCGGGAAATTATTTACCAAAGACTATGGTAAGGATTTTTCTGATCACCCA<br>CGGTATTACGTCAAAAGTCTCGACTCCTCAGCAGCTGGTGCATACCAGATTAAAAG<br>TTCCACATGGGACATGATTCTGAAGAAGTATAAGAAAACTTACGATATTACCGATT<br>TCTCACCAGCAAACCAGGATAAGGCTTGCCTTGTGCTGATTAAACACATTCGTAAT<br>GCACTTGACCTGATCGTGGACGAGAAGATTGATGAAGCCGTCCGGTCTCGTACCGA<br>TAATGATAAGAAACGCCTGCACTACGAATGGGCGTCAATGCCAGACTCTCCATATG<br>GGCAACGTACTATTACTATGGAGAAGTTTATGGAATACTACATGCATCATTTGGAG<br>CTCGAAGAGATTGGTATCTCGAACCTGGCCATCTCCAATAAAGACATCAAAAAATT<br>CCTCAATGGTTCGCATCATCATCACCATCATTAA

TRBA-Cly03	CCTGGATTTAATTATGGTGGAAAAGGTGATGGAACCGGCTGGAGCTCAGAACGTGG<br>GAGTGGTCCAGAGCCGGGTGGTGGTAGCCATGGAAATAGTGGTGGGCACGATCGT<br>GGAGATTCTTCCAACGTAGGTAATGAGTCTGTGACGGTAATGAAACCAGGGGATTC<br>GTATAACACCCCGTGGGGAAAAGTCATCATCAATGCTGCAGGCCAGCCGACCATGA<br>ACGGAACGGTGATGACCGCTGATAATTCATCGATGGTTCCTTACGGCAGAGGGTTT<br>ACACGGGTTTTAAATTCCCTGGTCAATAATCCTGTTTCGCCGGCAGGTCAGAATGG<br>CGGGAAGTCTCCTGTTCAGACTGCTGTGGAAAATTATCTGATGGTACAGTCAGGAA<br>ACCTGCCACCGGGCTACTGGCTCAGTAATGGCAAGGTTATGACGGAGGTTCGTGAG<br>GAACGTACTTCTGGCGGCGGTGGGAAAAACGGGAACGAGCGAACCTGGACTGTGA<br>AAGTTCCCCGGGAAGTACCTCAGCTTACGGCATCCTATAACGAGGGGATGAGAATC<br>CGACAGGAGGCAGCTGACCGTGCCAGAGCGGAAGCAAATGCCCGCGCTCTGGCTG<br>AAGAGGAAGCCCGTGCCATCGCATCAGGAAAGAGCAAAGCTGAGTTTGATGCAGG<br>TAAGCGGGTGGAGGCCGCACAGGCAGCGATTAATACAGCACAACTCAATGTTAAT<br>AACCTCAGCGGCGCTGTCAGTGCTGCAAATCAGGTTATAACTCAGAAACAGGCTGA<br>AATGACGCCCCTGAAAAATGAGCTTGCAGCCGCTAACCAGCGTGTCCAGGAGACG<br>CTTAAATTTATCAATGATCCTATTCGTAGCCGGATTCATTTTAATATGCGAAGTGGC<br>CTGATTCGCGCTCAACATAACGTTGATACTAAACAGAATGAAATTAATGCAGCAGT<br>GGCTAACCGTGATGCTCTGAATAGCCAATTGTCTCAGGCTAATAATATCCTGCAGA<br>ATGCCCGGAACGAAAAGAGTGCGGCTGATGCAGCACTTTCAGCTGCCACAGCACA<br>GCGGTTACAGGCAGAAGCCGCACTCAGGGCTGCTGCTGAGGCTGCAGAAAAAGCG<br>CGCCAGCGCCAGGCTGAAGGTGGAGGAGGATCCATGAATACGGACGCCTTGATTA<br>ACTGGTTTGAGAGCCGTCGCGGCAAACTCACTTATTCGATGTACGGGTCGCGTAAT<br>GGTTCTGATGGGACAGCAGATTGCAGCGGGTCGGTCAGTCAAGCGTTAAAGGAGG<br>CGGGGATTCCTATTAAAGGCCTTCCATCCACGGTTACTCTGGGCACCCAGCTCGCCC<br>AGAATGGCTTCATTCGGGTGTCACGGAATACTGACTGGACCGCTCAGCGGGGCGAC<br>ATCGTGCTTATGAGCTGGGGGGCTGATATGAGTACGTCCGGTGGCGCCGGTGGCCA<br>TGTGGGGGTAATGGAAGACGCTAACACTTTTATCAGCGTTGACTATTGGACGGGTG<br>GTGCGGCTGGTCAGGCTGTATCTTCCCACAATTGGGACCAGTATTACAACGCTCAA<br>CGTCCTCGCTATATCGAAGCATGGCGCTATAATGGCGCCGGGGGGAATCAACAGCC<br>TAACACCTCCGTTAGCCAAACGCCTACTCGTAAACCAGATTCTAAAGCATACTATC<br>TTGCTAATGACGTTGCTTTTGTTAACGGGATCTACCAGATTAAGTGCGATTACCTTG<br>CCCCGGTTGGTTTTGACTGGAACGATAATGGTGTGCCGGTTGGGCTGGTGAACTGG<br>GTCGACGAGAACGGCAATAATGTACGTGATGGCGCGGACAAGGACTTTAAACCAG

	GCATGTACTTCTCCTTTGAGATTGACGAGGCCCATATCGCCGATACAGGGGAGGGCGGCTATTATGGCGGGTATTACTGGCGCAAATTTGAATTCGGTCAATTCGGGACTGTGTGGCTTTCCTGTCGTGACAAGGATGATCTCGTAAACTACTACAAACATGGTTCGCATCATCATCACCATCATTAA
TRBK	GCAAAGGAACTCTCTGGTTACGGTCCAACTGCCGGCGAATCGATGGGTGGTACCGGGGCGAATCTCAACCAGCAGGGGGGCAACAACAACAGTAATTCAGGCGTTCATTGGGGTGGTGGGAGTGGTCACGGCAATAATGGCGGGCAGGGCAACAGTAATTCCTCTGGGTCCACCAGTACAGTGATGAAGACTGGCGAGTCGTATCTTACTCCTTGGGGTGATGTCGTCATCAACAATGACGGTTTGCCGGTAATGAATGGCATTGTTATGACGGAGGAAAACTCTACGCTCGTGGACAATCCGTTTGGCGGGGTGTCACGGGTGCTGAACTCACTGATCAGTGATATGCCTTCATTATTTGCTGAATCCTCTGGTAATAACAACAACAATACAGCATCAGTGAACACGGCCCCAACAAATGCGCAGGTGTCTGATATGGACAAGTCGTCCAAGGTCGTATCTAATGTAATTAATGAAAAGCAGAAGCAGAAGAATAAAATTGCAACTCAGATTTCGGAAAAACAGAAGAAGATTGAAGAGATGAAAAAGGTATTCAAACACCATTCATACCACGGGATTACCGATTTGGAGCGTGATGTGGATGAACTGCAGAAAAAATCTAACCAGTTAGATGCCGATATTTCAAAATTAAACTCGTACAAAAACACTTTACAGTCTAAAATTGGCGACGTGAACAAGCAGAAGGAAGCTGAAGAGAAAGCACGTGAAAATGCTGAAGTCGCAGAACACGAGACCCTGAACGAGGAGAAGCAAGCTGTCGCCGAGGCCGAGAAACGTCTCGCCGAGGCAAAAGCGGAGCTGGCGAAGGCCGAATCTGACGTGCAATCCAAACAGGCGACGGTATCGCGGGTAGCCGGTGAACTCGAAAATGCACAGAAATCCGTGGACGTCAAGGTAACCGGCTTTCCTGGCTGGCGGGACGTCCAAAAGAAATTAGGTTCGCATCATCATCACCATCATTAA
TRBK-Cly01	GCAAAGGAACTCTCTGGTTACGGTCCAACTGCCGGCGAATCGATGGGTGGTACCGGGGCGAATCTCAACCAGCAGGGGGGCAACAACAACAGTAATTCAGGCGTTCATTGGGGTGGTGGGAGTGGTCACGGCAATAATGGCGGGCAGGGCAACAGTAATTCCTCTGGGTCCACCAGTACAGTGATGAAGACTGGCGAGTCGTATCTTACTCCTTGGGGTGATGTCGTCATCAACAATGACGGTTTGCCGGTAATGAATGGCATTGTTATGACGGAGGAAAACTCTACGCTCGTGGACAATCCGTTTGGCGGGGTGTCACGGGTGCTGAACTCACTGATCAGTGATATGCCTTCATTATTTGCTGAATCCTCTGGTAATAACAACAACAATACAGCATCAGTGAACACGGCCCCAACAAATGCGCAGGTGTCTGATATGGACAAGTCGTCCAAGGTCGTATCTAATGTAATTAATGAAAAGCAGAAGCAGAAGAATAAAATTGCAACTCAGATTTCGGAAAAACAGAAGAAGATTGAAGAGATGAAAAAGGTATTCAAACACCATTCATACCACGGGATTACCGATTTGGAGCGTGATGTGGATGAACTGCAG

	AAAAAATCTAACCAGTTAGATGCCGATATTTCAAAATTAAACTCGTACAAAAACAC<br>TTTACAGTCTAAAATTGGCGACGTGAACAAGCAGAAGGAAGCTGAAGAGAAAGCA<br>CGTGAAAATGCTGAAGTCGCAGAACACGAGACCCTGAACGAGGAGAAGCAAGCTG<br>TCGCCGAGGCCGAGAAACGTCTCGCCGAGGCAAAAGCGGAGCTGGCGAAGGCCGA<br>ATCTGACGTGCAATCCAAACAGGCGACGGTATCGCGGGTAGCCGGTGAACTCGAA<br>AATGCACAGAAATCCGTGGACGTCAAGGTAACCGGCTTTCCTGGCTGGCGGGACGT<br>CCAAAAGAAATTAGGTGGAGGAGGATCCATGTGCTCAACAAAATCTATTGATCCTT<br>ACGGTGAGCAGAATAGTTATGATAATATTGGGTCAAAATACCAGAAAAATGATAC<br>GACCTCAGGTTACAATTTCAATTATAACCAGGCGGCTGACGAATTCTACAATATTA<br>ATCTGAGCAAGTACCTTAATAAGAAACTCGGGAACGATTGCTCGGGGTTTGTAAGC<br>TTAGTTAACGAAGACTCCAAGAGTCTGTATTTCGATGAGAACATTGTCAATAATTTT<br>TACGACAAGAACGGTCGCAAGTCCCAAGCTATCTTCAACCTTTATAAGTCCCAAAA<br>CAAAATTAGCTATACCGACCCTAAACCGGGCGATTTAGTATTTTTTAACAATACCA<br>CAAGCCGGACGAACAAATCTAAAAATAAGGCAATTACTCACCTCGGTATTATCGAC<br>AAAGTTGAGTCTAATGGTACTATCACGTTCGTTCACAACATTAACGGGAAGAATAT<br>CAAAAGCGTAATGAACTTGAAGCACAAGAACACACATAAATTAAATGGCAAGAAA<br>ATCAACGCTTATATTATTCTCAAGTGTCAAACGCCTGTATGTTTGATTTCCAACAAA<br>TTCGCTGGCTTTGGGAAAGTGGACAAATTTGTGCAAATTGATGGTTCGCATCACCA<br>CCACCACCATTAA
TRBK-Cly02	GCAAAGGAACTCTCTGGTTACGGTCCAACTGCCGGCGAATCGATGGGTGGTACCGG<br>GGCGAATCTCAACCAGCAGGGGGGCAACAACAACAGTAATTCAGGCGTTCATTGG<br>GGTGGTGGGAGTGGTCACGGCAATAATGGCGGGCAGGGCAACAGTAATTCCTCTG<br>GGTCCACCAGTACAGTGATGAAGACTGGCGAGTCGTATCTTACTCCTTGGGGTGAT<br>GTCGTCATCAACAATGACGGTTTGCCGGTAATGAATGGCATTGTTATGACGGAGGA<br>AAACTCTACGCTCGTGGACAATCCGTTTGGCGGGGTGTCACGGGTGCTGAACTCAC<br>TGATCAGTGATATGCCTTCATTATTTGCTGAATCCTCTGGTAATAACAACAACAATA<br>CAGCATCAGTGAACACGGCCCCAACAAATGCGCAGGTGTCTGATATGGACAAGTC<br>GTCCAAGGTCGTATCTAATGTAATTAATGAAAAGCAGAAGCAGAAGAATAAAATT<br>GCAACTCAGATTTCGGAAAAACAGAAGAAGATTGAAGAGATGAAAAAGGTATTCA<br>AACACCATTCATACCACGGGATTACCGATTTGGAGCGTGATGTGGATGAACTGCAG<br>AAAAAATCTAACCAGTTAGATGCCGATATTTCAAAATTAAACTCGTACAAAAACAC<br>TTTACAGTCTAAAATTGGCGACGTGAACAAGCAGAAGGAAGCTGAAGAGAAAGCA<br>CGTGAAAATGCTGAAGTCGCAGAACACGAGACCCTGAACGAGGAGAAGCAAGCTG

	TCGCCGAGGCCGAGAAACGTCTCGCCGAGGCAAAAGCGGAGCTGGCGAAGGCCGA
	ATCTGACGTGCAATCCAAACAGGCGACGGTATCGCGGGTAGCCGGTGAACTCGAA
	AATGCACAGAAATCCGTGGACGTCAAGGTAACCGGCTTTCCTGGCTGGCGGGACGT
	CCAAAAGAAATTAGGTGGAGGAGGATCCATGAACAACTTTATTAACTCTTTTAACG
	AATCCAAATCTATCACACGGGTGGATAAAGGCATGTGGGTCGATGGTGACAAAGG
	TGTTGGGGTGAAGGCCGATGGCGACATCCGTATTCTGCGTATTCGGGCGTTCATGT
	GCGCAATCAAATATGGGGAGGGCACTAGCGGCAACAACGGTTACGAAATCAACGT
	GGGCGGGAAATTATTTACCAAAGACTATGGTAAGGATTTTTCTGATCACCCACGGT
	ATTACGTCAAAAGTCTCGACTCCTCAGCAGCTGGTGCATACCAGATTAAAAGTTCC
	ACATGGGACATGATTCTGAAGAAGTATAAGAAAACTTACGATATTACCGATTTCTC
	ACCAGCAAACCAGGATAAGGCTTGCCTTGTGCTGATTAAACACATTCGTAATGCAC
	TTGACCTGATCGTGGACGAGAAGATTGATGAAGCCGTCCGGTCTCGTACCGATAAT
	GATAAGAAACGCCTGCACTACGAATGGGCGTCAATGCCAGACTCTCCATATGGGCA
	ACGTACTATTACTATGGAGAAGTTTATGGAATACTACATGCATCATTTGGAGCTCG
	AAGAGATTGGTATCTCGAACCTGGCCATCTCCAATAAAGACATCAAAAAATTCCTC
	AATGGTTCGCATCATCATCACCATCATTAA
TRBK-Cly03	GCAAAGGAACTCTCTGGTTACGGTCCAACTGCCGGCGAATCGATGGGTGGTACCGG
	GGCGAATCTCAACCAGCAGGGGGGCAACAACAACAGTAATTCAGGCGTTCATTGG
	GGTGGTGGGAGTGGTCACGGCAATAATGGCGGGCAGGGCAACAGTAATTCCTCTG
	GGTCCACCAGTACAGTGATGAAGACTGGCGAGTCGTATCTTACTCCTTGGGGTGAT
	GTCGTCATCAACAATGACGGTTTGCCGGTAATGAATGGCATTGTTATGACGGAGGA
	AAACTCTACGCTCGTGGACAATCCGTTTGGCGGGGTGTCACGGGTGCTGAACTCAC
	TGATCAGTGATATGCCTTCATTATTTGCTGAATCCTCTGGTAATAACAACAACAATA
	CAGCATCAGTGAACACGGCCCCAACAAATGCGCAGGTGTCTGATATGGACAAGTC
	GTCCAAGGTCGTATCTAATGTAATTAATGAAAAGCAGAAGCAGAAGAATAAAATT
	GCAACTCAGATTTCGGAAAAACAGAAGAAGATTGAAGAGATGAAAAAGGTATTCA
	AACACCATTCATACCACGGGATTACCGATTTGGAGCGTGATGTGGATGAACTGCAG
	AAAAAATCTAACCAGTTAGATGCCGATATTTCAAAATTAAACTCGTACAAAAACAC
	TTTACAGTCTAAAATTGGCGACGTGAACAAGCAGAAGGAAGCTGAAGAGAAAGCA
	CGTGAAAATGCTGAAGTCGCAGAACACGAGACCCTGAACGAGGAGAAGCAAGCTG
	TCGCCGAGGCCGAGAAACGTCTCGCCGAGGCAAAAGCGGAGCTGGCGAAGGCCGA
	ATCTGACGTGCAATCCAAACAGGCGACGGTATCGCGGGTAGCCGGTGAACTCGAA
	AATGCACAGAAATCCGTGGACGTCAAGGTAACCGGCTTTCCTGGCTGGCGGGACGT

CCAAAAGAAATTAGGTGGAGGAGGATCCATGAATACGGACGCCTTGATTAACTGG
TTTGAGAGCCGTCGCGGCAAACTCACTTATTCGATGTACGGGTCGCGTAATGGTTCT
GATGGGACAGCAGATTGCAGCGGGTCGGTCAGTCAAGCGTTAAAGGAGGCGGGGA
TTCCTATTAAAGGCCTTCCATCCACGGTTACTCTGGGCACCCAGCTCGCCCAGAATG
GCTTCATTCGGGTGTCACGGAATACTGACTGGACCGCTCAGCGGGGCGACATCGTG
CTTATGAGCTGGGGGGCTGATATGAGTACGTCCGGTGGCGCCGGTGGCCATGTGGG
GGTAATGGAAGACGCTAACACTTTTATCAGCGTTGACTATTGGACGGGTGGTGCGG
CTGGTCAGGCTGTATCTTCCCACAATTGGGACCAGTATTACAACGCTCAACGTCCTC
GCTATATCGAAGCATGGCGCTATAATGGCGCCGGGGGGAATCAACAGCCTAACAC
CTCCGTTAGCCAAACGCCTACTCGTAAACCAGATTCTAAAGCATACTATCTTGCTAA
TGACGTTGCTTTTGTTAACGGGATCTACCAGATTAAGTGCGATTACCTTGCCCCGGT
TGGTTTTGACTGGAACGATAATGGTGTGCCGGTTGGGCTGGTGAACTGGGTCGACG
AGAACGGCAATAATGTACGTGATGGCGCGGACAAGGACTTTAAACCAGGCATGTA
CTTCTCCTTTGAGATTGACGAGGCCCATATCGCCGATACAGGGGAGGGCGGCTATT
ATGGCGGGTATTACTGGCGCAAATTTGAATTCGGTCAATTCGGGACTGTGTGGCTTT
CCTGTCGTGACAAGGATGATCTCGTAAACTACTACAAAGGTTCGCATCATCATCAC
CATCATTAA

Table S3. Primers used in this study.

Primers	Primer sequence (5' to 3')
Cly01-F	CGCCATATGATGTGCTCAACAAAATCTATTGATCCT
Cly01-R	GCGTCGACTTAATGGTGGTGGTGGTGATGC
Cly02-F	CGCCATATGATGAACAACTTTATTAACTCTTTTAACG
Cly02-R	CCAAGCTTTTAATGATGGTGATGATGATG
Cly03-F	CGCCATATGATGAATACGGACGCCT
Cly03-R	CCAAGCTTTTAATGATGGTGATGATGATGCG
TRBA-F	CGCCATATGCCTGGATTTAATTATGGTGG
TRBA-R	CCAAGCTTTTAATGATGGTGATGATGATGCG
TRBK-F	CGCCATATGGCAAAGGAACTCTCTGGTTACG
TRBK-R	CCAAGCTTTTAATGATGGTGATGATGATGCGAACC
TRBA-Cly01-F	CGCCATATGCCTGGATTTAATTATGGTGGAAAAGG
TRBA-Cly01-R	GCGTCGACTTAATGGTGGTGGTGGTGATG
TRBA-Cly02-F	CGCCATATGCCTGGATTTAATTATGGTGGAAAAGG
TRBA-Cly02-R	CCAAGCTTTTAATGATGGTGATGATGATGCGAACC
TRBA-Cly03-F	CGCCATATGCCTGGATTTAATTATGGTGGAAAAGG
TRBA-Cly03-R	CCAAGCTTTTAATGATGGTGATGATGATGCGAACC
TRBK-Cly01-F	CGCCATATGGCAAAGGAACTCTCTGGTTACG
TRBK-Cly01-R	GCGTCGACTTAATGGTGGTGGTGGTGATG
TRBK-Cly02-F	CGCCATATGGCAAAGGAACTCTCTGGTTACG
TRBK-Cly02-R	CCAAGCTTTTAATGATGGTGATGATGATGCGAACC
TRBK-Cly03-F	CGCCATATGGCAAAGGAACTCTCTGGTTACG
TRBK-Cly03-R	CCAAGCTTTTAATGATGGTGATGATGATGCGAACC

The underlined region is the restriction site.

Table S4. Strains and plasmids used in this study.

Name	Relevant character(s)	Source
plasmids		
pET28 (a)	Expression vector, Kan^R	Novagen, Inc.
pPQL01	Cly01 gene cloned in pET28 (a) with NdeI and SalI	This study
pPQL02	Cly02 gene cloned in pET28 (a) with NdeI and HindIII	This study
pPQL03	Cly03 gene cloned in pET28 (a) with NdeI and HindIII	This study
pPQL04	TRBA gene cloned in pET28 (a) with NdeI and HindIII	This study
pPQL05	TRBA-Cly01 gene cloned in pET28 (a) with NdeI and SalI	This study
pPQL06	TRBA-Cly02 gene cloned in pET28 (a) with NdeI and HindIII	This study
pPQL07	TRBA-Cly03 gene cloned in pET28 (a) with NdeI and HindIII	This study
pPQL08	TRBK gene cloned in pET28 (a) with NdeI and HindIII	This study
pPQL09	TRBK-Cly01 gene cloned in pET28 (a) with NdeI and SalI	This study
pPQL10	TRBK-Cly02 gene cloned in pET28 (a) with NdeI and HindIII	This study
pPQL11	TRBK-Cly03 gene cloned in pET28 (a) with NdeI and HindIII	This study
Strains		
Escherichia coli		
PQL01	Applications strain of Cly01, containing plasmid pET28-Cly01 in DH5α	This study
PQL02	Applications strain of Cly02, containing plasmid pET28-Cly02 in DH5α	This study
PQL03	Applications strain of Cly03, containing plasmid pET28-Cly03 in DH5α	This study
PQL04	Applications strain of TRBA, containing plasmid pET28-TRBA in DH5α	This study
PQL05	Applications strain of TRBA-Cly01, containing plasmid pET28-TRBA-Cly01 in DH5α	This study
PQL06	Applications strain of TRBA-Cly02, containing plasmid pET28-TRBA-Cly02 in DH5α	This study
PQL07	Applications strain of TRBA-Cly03, containing plasmid pET28-TRBA-Cly03 in DH5α	This study
PQL08	Applications strain of TRBK, containing plasmid pET28-TRBK in DH5α	This study
PQL09	Applications strain of TRBK-Cly01, containing plasmid pET28-TRBK-Cly01 in DH5α	This study

PQL10	Applications strain of TRBK-Cly02, containing plasmid pET28-TRBK-Cly02 in DH5α	This study
PQL11	Applications strain of TRBK-Cly03, containing plasmid pET28-TRBK-Cly03 in DH5α	This study
PQL12	Expression strain of Cly01, containing plasmid pET28-Cly01 in BL21 (DE3)	This study
PQL13	Expression strain of Cly02, containing plasmid pET28-Cly02 in BL21 (DE3)	This study
PQL14	Expression strain of Cly03, containing plasmid pET28-Cly03 in BL21 (DE3)	This study
PQL15	Expression strain of TRBA, containing plasmid pET28-TRBA in BL21 (DE3)	This study
PQL16	Expression strain of TRBA-Cly01, containing plasmid pET28-TRBA-Cly01 in BL21 (DE3)	This study
PQL17	Expression strain of TRBA-Cly02, containing plasmid pET28-TRBA-Cly02 in BL21 (DE3)	This study
PQL18	Expression strain of TRBA-Cly03, containing plasmid pET28-TRBA-Cly03 in BL21 (DE3)	This study
PQL19	Expression strain of TRBK, containing plasmid pET28-TRBK in BL21 (DE3)	This study
PQL20	Expression strain of TRBK-Cly01, containing plasmid pET28-TRBK-Cly01 in BL21 (DE3)	This study
PQL21	Expression strain of TRBK-Cly02, containing plasmid pET28-TRBK-Cly02 in BL21 (DE3)	This study
PQL22	Expression strain of TRBK-Cly03, containing plasmid pET28-TRBK-Cly03 in BL21 (DE3)	This study
DH5α	F^-, φ80d/*lacZ*ΔM15, Δ(*lacZYA-argF*) U169 *recA*1 *endA*1 *hsdR*17	Laboratory collection
BL21	F^-, *ompT hsdS$_B$* (r_B^-, m_B^-) *gal dcm* (DE3)	Laboratory collection
MG1655	F_- λ_- *ilvG*$_-$ *rfb-50 rph-1*	Laboratory collection
M12	Clinical isolates, wild type, H11	Laboratory

		collection
M16	Clinical isolates, wild type, O116:H32	Laboratory collection
W31	Clinical isolates, wild type, O117:H42	Laboratory collection
W79	Clinical isolates, wild type, O16:H48	Laboratory collection
Salmonella enteritidis		
CVCC3377	Wild type strain	China veterinary culture collection
Salmonella typhimurium		
A13	Clinical isolates, wild type	Laboratory collection
Campylobacter jejuni		
E62	Clinical isolates, wild type	Laboratory collection
KT-32	Clinical isolates, wild type	Laboratory collection

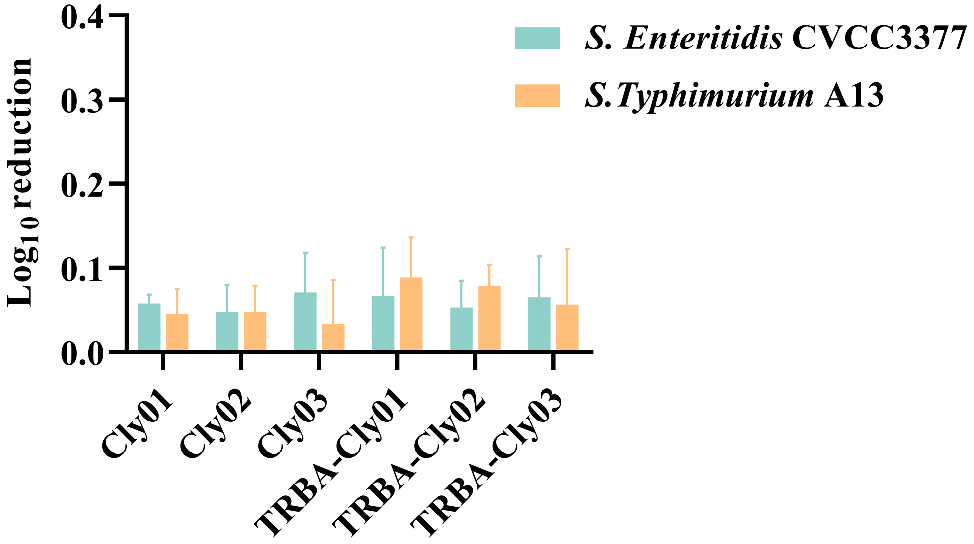

Fig. S1. Antibacterial activity test of Clysins and TRBA-Clysins against *Salmonella* spp. Antibiotic bioactivity of Clysins and TRBK-Clysins against logarithmic *Salmonella Enteritidis* CVCC3377 and *Salmonella Typhimurium* A13 strain under the concentration of 1 μM (24.8 μg/mL for Cly01, 25.6 μg/mL for Cly02, 32.2 μg/mL for Cly03, 64.9 μg/mL for TRBA-Cly01, 65.8 μg/mL for TRBA-Cly02 and 72.6 μg/mL for TRBA-Cly03) in 10 mM PBS (pH 7.4) depicted in $\log_{10}$ CFU reduction**.** The data are presented as the mean ± standard deviation of triplicate independent experiments

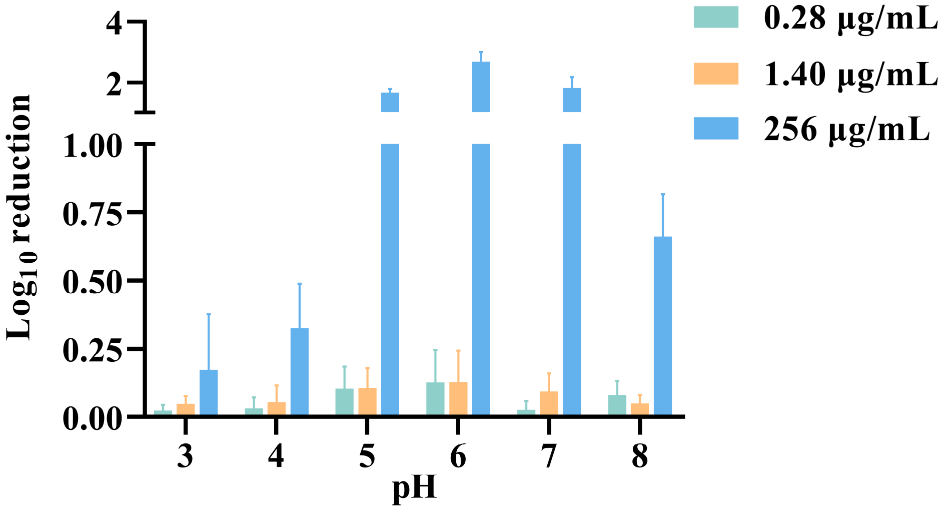

Fig. S2. Effects of pH on the activity of Cly02 against *E. coli* BL21 in PBS. Antibiotic bioactivity of Cly02 against logarithmic *E. coli* BL21 strain under the concentration of 0.28 μg/mL 1.40 μg/mL and 256 μg/mL in 10 mM PBS at pH 3-8 depicted in $\log_{10}$ CFU reduction**.** The data are presented as the mean ± standard deviation of triplicate independent experiments

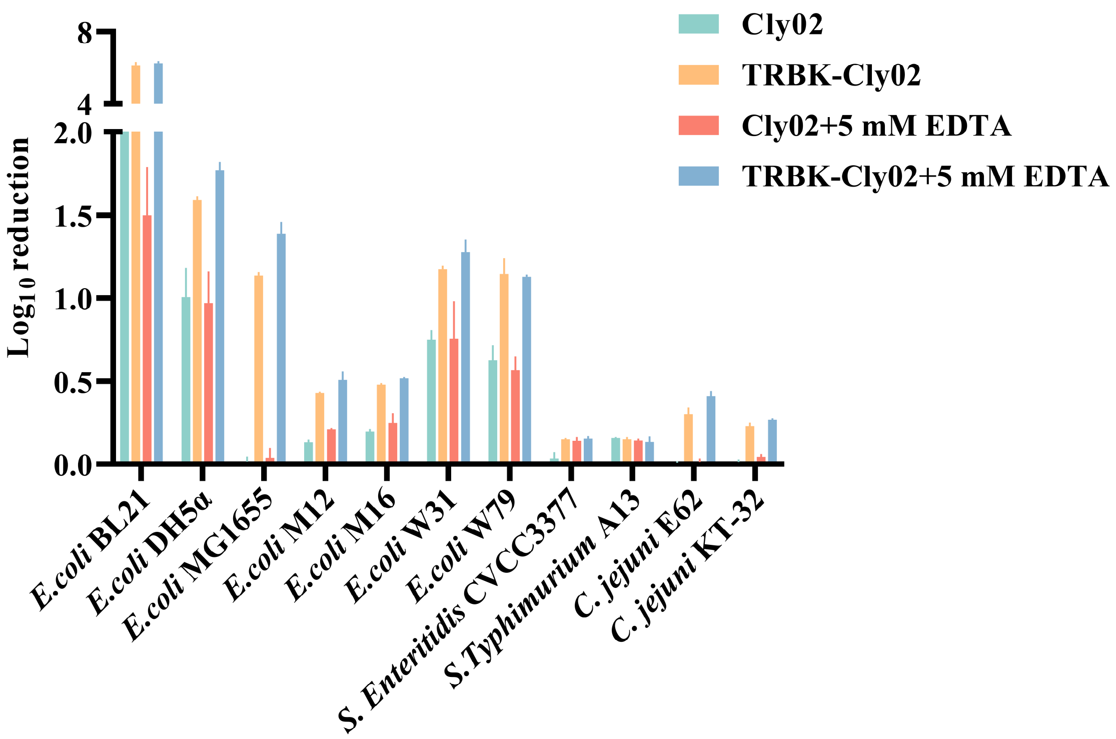

Fig. S3. Antimicrobial spectrum of Cly02 and TRBK-Cly02 against *E. coli*, *Salmonella* spp., and *Campylobacter jejuni* strains. The antibacterial activities of 25.63 µg/mL Cly02 and 60.10 µg/mL TRBK-Cly02 were assessed in PBS (pH 6.0) with or without 5 mM EDTA and indicated by log_{10} reduction of cells. Data are presented as the mean ± standard deviation of triplicate independent experiments.

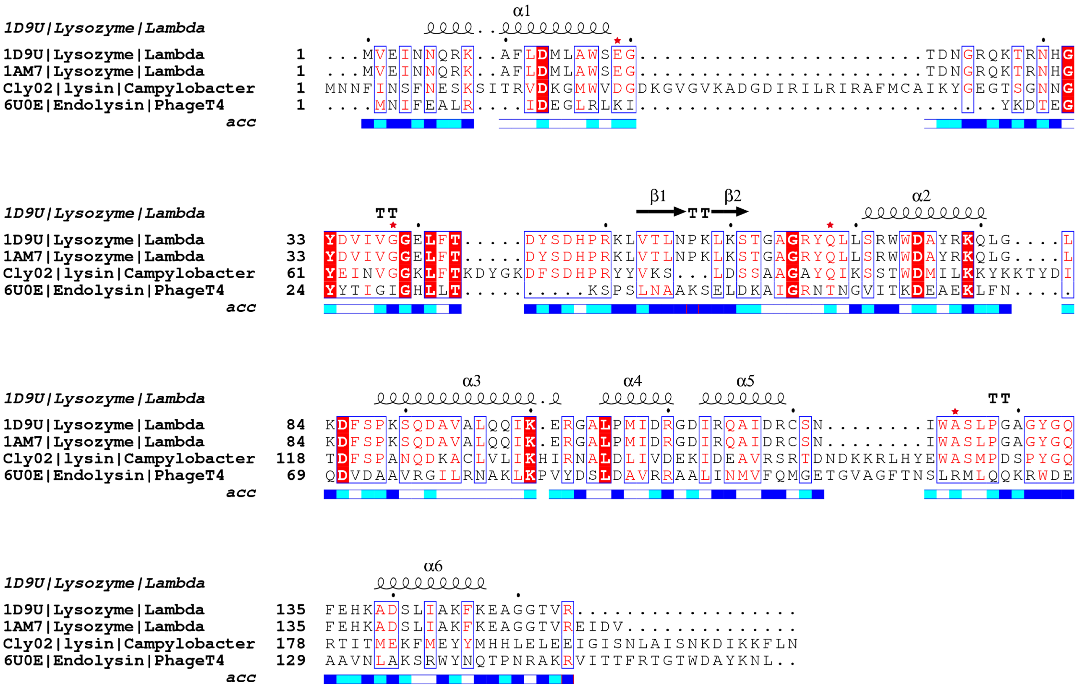

Fig. S4. Sequence analysis of Cly02 with lytic transglycosylase, lambda lysozyme and *Escherichiavirus* T4 endolysin. The red background represents the same amino acid residue, and the blue square represents the residue region with a high identity. The key amino acid sites of 1D9U have been marked with red stars.
